# Loss of the Chr16p11.2 ASD candidate gene *QPRT* leads to aberrant neuronal differentiation in the SH-SY5Y neuronal cell model

**DOI:** 10.1186/s13229-018-0239-z

**Published:** 2018-11-06

**Authors:** Denise Haslinger, Regina Waltes, Afsheen Yousaf, Silvia Lindlar, Ines Schneider, Chai K. Lim, Meng-Miao Tsai, Boyan K. Garvalov, Amparo Acker-Palmer, Nicolas Krezdorn, Björn Rotter, Till Acker, Gilles J. Guillemin, Simone Fulda, Christine M. Freitag, Andreas G. Chiocchetti

**Affiliations:** 1 Department of Child and Adolescent Psychiatry, Psychosomatics and Psychotherapy, University Hospital Frankfurt, JW Goethe University Frankfurt, Frankfurt am Main, Germany; 2 Institute of Experimental Cancer Research in Pediatrics, Frankfurt am Main, Germany; 3 Faculty of Medicine and Health Sciences, Macquarie University, Sydney, New South Wales Australia; 4 Neuropathology, University of Giessen, Giessen, Germany; 5 Department of Microvascular Biology and Pathobiology, European Center for Angioscience (ECAS), Medical Faculty Mannheim, University of Heidelberg, Heidelberg, Germany; 6 Institute of Cell Biology and Neuroscience and Buchmann Institute for Molecular Life Sciences (BMLS), JW Goethe University of Frankfurt, Frankfurt am Main, Germany; 7 GenXPro GmbH, Frankfurt am Main, Germany

**Keywords:** Autism, 16p11.2, Quinolinate phosphoribosyltransferase, Quinolinic acid, Kynurenine, CRISPR/Cas9, Sholl analysis

## Abstract

**Background:**

Altered neuronal development is discussed as the underlying pathogenic mechanism of autism spectrum disorders (ASD). Copy number variations of 16p11.2 have recurrently been identified in individuals with ASD. Of the 29 genes within this region, *quinolinate phosphoribosyltransferase* (*QPRT*) showed the strongest regulation during neuronal differentiation of SH-SY5Y neuroblastoma cells. We hypothesized a causal relation between this tryptophan metabolism-related enzyme and neuronal differentiation. We thus analyzed the effect of *QPRT* on the differentiation of SH-SY5Y and specifically focused on neuronal morphology, metabolites of the tryptophan pathway, and the neurodevelopmental transcriptome.

**Methods:**

The gene dosage-dependent change of *QPRT* expression following Chr16p11.2 deletion was investigated in a lymphoblastoid cell line (LCL) of a deletion carrier and compared to his non-carrier parents. Expression of *QPRT* was tested for correlation with neuromorphology in SH-SY5Y cells. QPRT function was inhibited in SH-SY5Y neuroblastoma cells using (i) siRNA knockdown (KD), (ii) chemical mimicking of loss of QPRT, and (iii) complete CRISPR/Cas9-mediated knock out (KO). *QPRT*-*KD* cells underwent morphological analysis. Chemically inhibited and *QPRT-KO* cells were characterized using viability assays. Additionally, *QPRT-KO* cells underwent metabolite and whole transcriptome analyses. Genes differentially expressed upon KO of *QPRT* were tested for enrichment in biological processes and co-regulated gene-networks of the human brain.

**Results:**

*QPRT* expression was reduced in the LCL of the deletion carrier and significantly correlated with the neuritic complexity of SH-SY5Y. The reduction of *QPRT* altered neuronal morphology of differentiated SH-SY5Y cells. Chemical inhibition as well as complete KO of the gene were lethal upon induction of neuronal differentiation, but not proliferation. The QPRT-associated tryptophan pathway was not affected by KO. At the transcriptome level, genes linked to neurodevelopmental processes and synaptic structures were affected. Differentially regulated genes were enriched for ASD candidates, and co-regulated gene networks were implicated in the development of the dorsolateral prefrontal cortex, the hippocampus, and the amygdala.

**Conclusions:**

In this study, *QPRT* was causally related to in vitro neuronal differentiation of SH-SY5Y cells and affected the regulation of genes and gene networks previously implicated in ASD. Thus, our data suggest that *QPRT* may play an important role in the pathogenesis of ASD in Chr16p11.2 deletion carriers.

**Electronic supplementary material:**

The online version of this article (10.1186/s13229-018-0239-z) contains supplementary material, which is available to authorized users.

## Background

Altered neuronal development is suggested to be one of the major drivers in the etiology of autism spectrum disorders (ASD). Neuropathological studies based on postmortem brains of ASD patients reported aberrant neuronal development including reduced dendritic branching in the hippocampus [1], smaller pyramidal neurons in the language associated Broca’s area [2], abnormal minicolumnar organization in the cerebral cortex leading to decreased inter-areal connectivity [3], and disorganized layers of the cortical areas [4]. The underlying etiology of ASD is mainly based on different genetic findings including de novo copy number variations (CNVs). These CNVs, in particular deletions, have been recurrently shown to alter genic regions in ASD individuals [5], specifically affecting neurodevelopmental genes [6].

One of the most recurrent CNVs in ASD resides within Chr16p11.2 spanning ~ 600 kb. Overall, duplications and deletions of 16p11.2 can be identified in 0.8% of ASD cases [7]. A deletion of this region is associated with a nine times higher likelihood of developing ASD, and the duplication is associated with a nine times higher risk of both ASD and schizophrenia [8]. While developmental delay or intellectual disability can occur in some cases of 16p11.2 duplication carriers, they are more common in deletions [9, 10].

The 16p11.2 CNV region spans 29 genes which showed gene dosage-dependent expression in lymphoblastoid cell lines (LCLs) of CNV carriers, leading to a differential expression of genes implicated in biological processes such as synaptic function or chromatin modification [11].

A study in zebrafish showed that the majority of the human Chr16p11.2 homologous genes are involved in nervous system development: loss of function of these genes led to an altered brain morphology for 21 of 22 tested genes [12]. Double heterozygous knockouts of the Chr16p11.2 homologs *double C2 domain alpha* (*DOC2A*) and *family with sequence similarity 57, member Ba* (*FAM57BA*) induced hyperactivity, increased seizure susceptibility and increased body length and head size in zebrafish [13]. In mice, CNVs of the homologous 16p11.2 region induced differing phenotypes. Two studies reported the deletion to result in a reduction of the skull [14] or brain size [15], accompanied by gene dosage-sensitive changes of behavior and synaptic plasticity [14] as well as altered cortical cytoarchitecture, and reduction of downstream extracellular signaling-related kinase (ERK/MAPK) effectors [15]. Comparing individual brain regions in mice with deletions to wild-type animals, Horev and associates identified six regions with an increased volume, which were not altered in duplication carriers (see Dataset S04 in the original publication [16]). In another study, mice carrying a heterozygous microduplication of the region showed increased dendritic arborization of cortical pyramidal neurons [17]. Via network analysis of protein-protein-interaction, the authors identified the gene coding for mitogen-activated protein kinase 3 (MAPK3) as hub gene. MAPK3 plays a role in signaling cascades involved in proliferation and differentiation. A recent study focused on different effects of 16p11.2 deletions in male and female mice and reported impairments of reward-directed learning in male mice accompanied by male-specific overexpression of *dopamine receptor D2* (*DRD2*) and *adenosine receptor 2a* (*ADORA2A*) in the striatum [18]. Both genes have been discussed in the context of ASD [19, 20].

While the functional validation of the entire CNV models the genomic status of the patients, investigating gene dosage effects of single genes located in Chr16p11.2 is useful to understand their individual contribution to the complex and diverse pathologies of ASD. In zebrafish, the suppression of *potassium channel tetramerization domain containing 13 (KCTD13*) was associated with macrocephaly whereas overexpression led to microcephaly [21]. In mice, the same study showed a reduction of *KCTD13* to result in increased proliferation of neuronal progenitors, which is also suggested to result in macrocephaly. Further, a heterozygous deletion of the gene coding for major vault protein (*MVP*) induced a reduction of functional synapses in mice [22]. *TAO kinase 2 (TAOK2*), also located in 16p11.2, was found to be essential for the development of basal dendrites and axonal projections in cortical pyramidal neurons of mice [23]. Chr16p11.2 genes *DOC2A*, *KIF22*, and *T-box 6* (*TBX6*) are required for the development of neuronal polarity in mouse hippocampal cultures [24].

In ASD patients, multiple brain measures such as the thalamic or total brain volume were reported to be increased in 16p11.2 deletion carriers and reduced in duplication carriers [25, 26]. Another study integrated physical interactions of 16p11.2 proteins with spatiotemporal gene expression of the human brain. The authors identified the KCTD13-Cul3-RhoA pathway as being crucial for controlling brain size and connectivity [27]. Still, only few genes of the Chr16p11.2 region have been investigated for their specific role in neuronal differentiation in human models.

Here, we investigated the SH-SY5Y neuroblastoma cell model as a well-studied and feasible model for neuronal differentiation in vitro. Previously, we reported that a continuous application of brain-derived neurotrophic factor (BDNF) and retinoic acid (RA) leads to neuronal cells of most likely cortical identity with a transcriptomic signature reminiscent of that of neocortical brain tissue developed for 16–19 weeks post-conception [28]. In addition, we showed expressed genes in the differentiated SH-SY5Y model to be co-regulated within modules, several of which were associated with neurodevelopmental disorders such as the orange module. We then implemented three complementary statistical methods to identify genes that were (i) differentially regulated upon differentiation, (ii) significantly involved in the independent processes active during differentiation and/or (iii) that were significantly changed over time. Finally, we described a list of 299 robustly regulated genes that appeared to be significant in all three analyses [28].

We here report that of the 29 genes located within Chr16p11.2, a total of 10 genes were identified by at least one of the three implemented statistical approaches. However, only the gene coding for *quinolinate phosphoribosyltransferase* (*QPRT)* was identified by all three analyses. In addition, *QPRT* was one of the most highly expressed genes of the Chr16p11.2 region and showed the highest regulatory fold change (FC) after induction of neuronal differentiation. Also, *QPRT* was co-regulated with an early upregulated gene module (MEorange) which showed significant enrichment for ASD candidate genes [28].

*QPRT* codes for an enzyme of the kynurenine pathway, the primary route for tryptophan catabolism, which results in the production of nicotinamide adenine dinucleotide (NAD^+^). In addition, it is the only enzyme catabolizing quinolinic acid (QUIN), a potent excitotoxin acting as N-methyl-D-aspartate receptor (NMDA-R) agonist. QUIN is also linked to astroglial activation and cell death as originally identified in the context of Alzheimer’s disease [29]. *QPRT-KO* mice showed increased QUIN levels in the brain [30] and increased excretion of QUIN in urine [31]. A significant increase of QUIN was observed in blood plasma of children with ASD when compared to their age-matched healthy control siblings [32]. Furthermore, QPRT was identified as an interaction partner of the ASD candidate neuroligin 3 (NLGN3; [33]), suggesting an involvement of QPRT in the formation of the postsynaptic density.

Here, we hypothesized that *QPRT* is implicated in neuronal differentiation and that reduced *QPRT* expression following its deletion results in alterations of neuromorphological development. We first tested the gene dosage-dependent expression of *QPRT* in a patient-specific LCL of one Chr16p11.2 deletion carrier. We then analyzed the expression of *QPRT* and its co-regulated gene set for correlation with the development of neuronal morphology in SH-SY5Y wild-type (WT) cells. To study the effects on neuronal morphology, we inhibited QPRT function in SH-SY5Y cells using (i) siRNA knockdown (KD), (ii) chemical mimicking of loss of QPRT, and (iii) complete CRISPR/Cas9-mediated knock out (KO). *QPRT-KD* cells underwent morphological analysis. Chemically inhibited and *QPRT-KO* cells were characterized using viability assays. To understand the effects of QPRT loss on the kynurenine pathway and QUIN levels, we additionally performed a metabolite analysis of the generated *QPRT-KO* cells. To explore the systems-wide interaction network of QPRT, we investigated the transcriptomic signature of *QPRT-KO* cells. Finally, to understand the role of *QPRT* in neural development, we tested the genes associated with *QPRT-KO* for enrichment among gene-networks implicated in human brain development [34].

## Methods

### Cellular models

#### Patient-specific lymphoblastoid cell lines (LCLs)

Lymphoblastoid cell lines (LCLs) were generated as previously published [35, 36]. We investigated LCLs generated from one Chr16p11.2 heterozygous deletion carrier and his non-carrier parents. The child was diagnosed with autism (ICD10: F84.0) based on both the Autism Diagnostic Interview-Revised (ADI-R; [37, 38]) and the Autism Observation Schedule (ADOS; [39]). The patient presented with a severe impairment of social interaction, hyperactive and aggressive behavior as well as language delay, and an average non-verbal IQ = 90. The deletion was identified in a screen of 710 children with ASD and their parents using the Illumina Human OmniExpress Microarray and validated via real-time PCR (unpublished data). To investigate *QPRT* gene expression at transcriptional level in exponentially growing cell cultures of the Chr16p11.2 deletion carrier compared to his non-carrier parents, 1 × 10^6^ viable cells were inoculated into 5 ml RPMI medium supplied with 10% fetal bovine serum (FBS), 100 U/ml penicillin, 100 μg/ml streptomycin, 1X GlutaMAX (all from GIBCO®, Life Technologies, Paisley, UK). Volume was doubled when cultures reached a density of 1 × 10^6^ cells/ml. Finally, cells were harvested with a total volume of 20 ml at a density of again 1 × 10^6^ cells/ml. Whole RNA was extracted, including DNase treatment, and reversely transcribed using the GeneJet RNA Purification Kit and the RevertAid H Minus first strand cDNA synthesis kit (both from Thermo Fisher Scientific) following the manufacturer’s protocols. Real-time RT-PCR was performed using the Universal Probe Library (UPL; Roche) and a StepOne Plus device (Applied Biosystems) and normalized to *glucuronidase beta* (*GUSB*; see Additional file 1: Supplementary Methods and Table S1).

#### Neuroblastoma cell line SH-SY5Y

Out of possible cell types to investigate neuronal differentiation, SH-SY5Y is well studied, feasible, and with an optimized protocol shows reproducible differentiation of cells into cortical-like neurons [28]. For proliferation, cells were cultured in Dulbecco’s modified Eagle’s medium (DMEM) supplemented with 10% FBS, 1% sodium pyruvate (all from Life Technologies), and 1% penicillin/streptomycin (PAA). As described in our previous study [28], SH-SY5Y cells were differentiated using a continuous application of retinoic acid (RA) and brain-derived neurotrophic factor (BDNF). Differentiation media consisted of Neurobasal®-A medium supplemented with 1x GlutaMAX, 1x B-27 supplement (all from Life Technologies), 10 μM RA, 2 mM cAMP (both from Sigma-Aldrich), 50 ng/ml hBDNF (Immunotools), 1% penicillin/streptomycin (PAA), and 20 mM KCl. Cells were differentiated for 11 days changing the medium every other day. The time points for extraction of mRNA or protein and imaging were set 24 h after media changes (0/undifferentiated cells, 1, 3, 5, 7, 9, and 11 days of in vitro differentiation).

To study the morphological development of WT SH-SY5Y cells during neuronal differentiation, we transfected proliferating cells with pmaxGFP (Lonza) using Metafectene Pro (Biontex) according to the manufacturer’s protocol. One day after transfection, cells were seeded 1:2 in co-cultures with untransfected cells with a density of 1 × 10^4^ cells/cm^2^ to allow imaging of individual transfected cells. Cultures were imaged using a Motic AE31 fluorescence microscope (Motic). Images were analyzed using custom macros in ImageJ [40] available on request. In short, all images were equalized, despeckled, and the background was subtracted using the rolling ball method prior to binarization (Auto threshold “Otsu-dark”). Sholl analysis [41], a concentric circle method, was performed using the respective ImageJ plugin with manual selection of the cells’ center and with fitting polynomial regression of the fifth degree, as suggested by the manual [42]. The following morphological parameters were assessed: maximum intersections (maximum number of neuritic intersections for one radius), sum of intersections (sum of all intersections of one cell), enclosing radius (the outer radius intersecting the cell, describing the longest distance between soma and neurites), intersecting radii (number of radii intersecting a cell, also describing the distance between soma and neurites), average number of intersections (the number of intersections analyzed divided by the number of intersecting radii; a measure describing neuritic complexity) and maximum intersections radius (the distance from the soma where most neurites are present, i.e., the site of the highest branching density; also reflecting neuritic complexity). Morphological parameters have been assessed in parallel during the previous transcriptomic analysis [28].

We made use of the previously published whole transcriptome data of differentiating SH-SY5Y cells (gene expression omnibus repository (GEO) under the accession number GSE69838 [28]) and analyzed *QPRT* expression as well as regulation of modules of co-expressed genes for correlation with the simultaneously assessed morphological parameters of SH-SY5Y neuronal differentiation (see also the “Statistical analysis” section below). Furthermore, *QPRT* expression during neuronal differentiation was validated in the same mRNA extracts using real-time RT-PCR. RNA extraction, cDNA generation, and real-time RT-PCR were performed as described above and in Additional file 1: Supplementary Methods. Expression values were normalized to *GUSB* and *glyceraldehyde-3-phosphate dehydrogenase* (*GAPDH*).

### Reduction of QPRT in neuroblastoma cell line SH-SY5Y

#### siRNA-mediated knockdown (KD) of QPRT

QPRT expression in SH-SY5Y was reduced using three different siRNAs (siQ1–siQ3) and compared to a non-targeting control siRNA (siCtrl) using the Ambion Silencer Select siRNAs (Thermo Fisher; Additional file 1: Supplementary Methods). siRNA-generated KD of QPRT after 11 days of differentiation was proven in parallel to the morphological analysis on protein level via Western Blot as described in Additional file 1: Supplementary Methods. In short, QPRT (mouse anti-QPRT, Abcam) and control β-Actin (ACTB; mouse anti-β-Actin, Sigma) were detected using the secondary antibody anti-mouse IgG HRP-conjugated (Santa Cruz), the ECL Prime Western Blot Detection Reagent, and the Amersham Hyperfilm ECL (both from GE Healthcare). To allow imaging of fluorescent single cells in order to study morphological effects of *QPRT* knockdown, we co-transfected SH-SY5Y cells with mCherry (Plasmid #30125; Addgene). In a 96-well format, 5.2 × 10^4^ cells/cm^2^ were reversely transfected with 120 ng of mCherry and 5 nM siRNA using Lipofectamine RNAi Max and OptiMEM (Thermo Fisher).

#### CRISPR/Cas9 mediated knock out (KO) of QPRT

CRISPR/Cas9 gene editing was performed as described elsewhere [43]. In short, sgRNAs were designed [44] and oligos were ordered from Sigma-Aldrich and cloned into pSpCas9(BB)-2A-Puro (PX459) V2.0 (Plasmid #62988; Addgene). Two sgRNAs targeting different sequences of *QPRT* were designed to generate homozygous knock out in addition to the non-targeting empty control vector (see Additional file 1: Supplementary Methods and Supplementary Results). Validated plasmids (Sanger sequencing) were transfected into SH-SY5Y cells using Metafectene Pro (Biontex) according to the manufacturer’s protocol with subsequent puromycin (Sigma-Aldrich) selection using 750 ng/ml for 7 to 14 days. After low-density seeding, single clones were isolated using 5 μl of Trypsin-EDTA (Thermo Fisher Scientific) and expanded in 96-well plates. Overall, we sequenced at least ten clones per construct and confirmed homozygous single cell clones at least twice by Sanger sequencing including the empty control vector. Clones were named as follows: del268T (*QPRT* NM_014298 del268T; Ex2.1), ins395A (*QPRT* NM_014298 ins395A; Ex2.2), eCtrl (empty control vector). Both indels induced premature stop codons as validated in silico and by Sanger sequencing. KO of *QPRT* was further shown on RNA and protein level using real-time RT-PCR and Western Blot, respectively. RNA extraction, cDNA generation, and real-time RT-PCR as well as protein extraction and Western Blots were performed as described above and in Additional file 1: Supplementary Methods. RNA expression values were normalized to *GUSB* and *GAPDH*, and protein expression was descriptively compared to GAPDH expression.

### Functional analyses in neuroblastoma cell line SH-SY5Y

#### siRNA-mediated KD of QPRT

The day after transfection (see above), media were changed from proliferation to differentiation media. After changing the media every other day for a time course of 11 days, cells were imaged using an ImageXPress Micro XLS (Molecular Devices). Images were pre-processed in MetaXPress and analyzed in ImageJ using Sholl analysis (as described above).

#### Chemical mimicking of QPRT loss

To measure the effect of chemical QPRT inhibition, we seeded WT SH-SY5Y cells with a density of 2.5 × 10^4^ cells/cm^2^. After cells have attached overnight, media were changed to either fresh proliferation or differentiation media containing 0 (reference), 5, or 10 mM of the QPRT inhibitor phthalic acid (PA; Sigma; directly diluted in the respective media; [45]) and incubated for 3 days followed by propidium iodide (PI) viability assays. PI and Hoechst33342 (both from Sigma) were pre-diluted in DPBS (Life Technologies) and used with final concentrations of 1 μg/ml and 10 μg/ml for the assay, respectively. Prior to analysis, supernatant including detached and dead cells was removed and attached cells were washed with DPBS. Next, attached cells were incubated with PI and Hoechst33342 for 5 min at 37 °C and imaged using the ImageXPress Micro XLS microscope (Molecular Devices). Images were acquired through DAPI and TRITC channels and analyzed using the MetaXPress macro “Cell Scoring”. Percentage of dead cells after application of 5 and 10 mM PA was compared to the 0 mM reference for proliferating and differentiating cells, respectively.

To test if the observed cell death upon chemical inhibition of QPRT during differentiation is driven by an accumulation of the QPRT substrate quinolinic acid (QUIN; Sigma), we exposed WT cells to this neurotoxin. Cells were seeded with a density of 2.5 × 10^4^ cells/cm^2^ and media were changed after 24 h to either fresh proliferation or differentiation media containing 0 (reference; vehicle H_2_O only), 5, or 250 μM of QUIN [46] and incubated for 3 days followed by PI viability-assays (as described above). Percentage of dead cells after application of 5 and 250 μM QUIN was compared to the 0 μM reference for proliferating and differentiating cells, respectively.

#### CRISPR/Cas9-mediated KO of QPRT

##### Viability assay

To measure the effect of *QPRT-KO* on the viability of differentiating SH-SY5Y, KO and eCtrl cells were seeded at a density of 2.5 × 10^4^ cells/cm^2^. The following day, media were changed to differentiation media. After 3 days, PI viability-assays were performed (as described above).

##### Rescue experiments of *QPRT-KO* cells

We further aimed to rescue the effects of a potential *QPRT-KO*-driven increase of QUIN by inhibiting downstream pathways. Cells were seeded with a density of 2.5 × 10^4^ cells/cm^2^. After 24 h, media were changed to differentiation media containing (i) 0 (reference; vehicle H_2_O only), 6, and 12 μM of the NMDA-R antagonist MK801 [47], (ii) 0 (reference; vehicle H_2_O only), 0.5, and 1 mM of nitric oxide synthase 1 (NOS1) inhibitor L-NAME [48], and (iii) 0 (reference; vehicle H_2_O only), 5, and 10 mM of NAD^+^ [49]. PI viability assays were performed after an incubation of 3 days (as described above). All rescue experiments were performed with differentiating *QPRT-KO* cell lines compared to the eCtrl.

##### Metabolite analysis

To characterize *QPRT-KO* at the level of the kynurenine pathway, i.e., tryptophan catabolism, we analyzed its metabolites in cell culture supernatants using ultra-high-performance liquid chromatography (UHPLC; for tryptophan (TRP), kynurenine (KYN), 3-hydroxykynurenine (3HK), 3-hydroxyanthranilic acid (3HAA), anthranilic acid (AA) and kynurenic acid (KA)), and gas chromatography-mass spectrometry (GC/MS; for picolinic acid (PIC) and QUIN). Cell lines were seeded with a density of 2 × 10^4^ cells/cm^2^ and grown in proliferation or differentiation media for 3 days as described above. Supernatants of replicates were harvested and stored at − 80 °C until further proceedings. UHPLC and GC/MS were performed as described previously [32, 50] (see also Additional file 1: Supplementary Methods).

##### Massive analysis of cDNA ends (MACE)

To investigate the transcriptomic changes induced by *QPRT-KO*, we performed a whole transcriptome analysis using the RNA-Seq approach MACE. MACE sequencing in contrast to RNA sequencing reads cDNA ends only rather than whole transcripts, i.e., poly-A tails. The output values of MACE-Seq are absolute counts per cDNA end. This approach is more sensitive compared to classical RNA-Seq. This method allows the detection of low abundant transcripts and differentiation between alternative 3′UTRs with high accuracy [51, 52]. However, it does not allow to identify alternative exon usage.

Cell lines (three biological replicates of each WT, eCtrl, and KO cells) were seeded with a density of 2 × 10^4^ cells/cm^2^ in proliferation medium. After 3 days, media were changed to differentiation and cells were differentiated for 3 days without media changes. RNA was prepared as described above. RNA integrity number (RIN) was analyzed using the LabChip GX system and only samples with RINs above 9.7 underwent further analysis. MACE analysis including quality control was outsourced to GenXPro (Frankfurt) [53]. Reads were mapped to the human genome version hg38 using the Bowtie2 algorithm implementing the “--sensitive-local” parameter and standard settings as published [54].

A total of 12 genes found to be significantly differentially expressed in KO cells with an absolute log2 fold change (FC) above 2.5 were chosen for validation via real-time RT-PCR using the UPL system as described above with *GAPDH*, *GUSB*, and *proteasome 26S subunit, non-ATPase 7* (*PSMD7*) as housekeeping genes. Additionally, the GOIs *QPRT*, *nicotinamide nucleotide adenylyltransferase 2* (*NMNAT2*; only downstream enzyme of *QPRT* differentially regulated upon KO of *QPRT*), and *NLGN3* were included for validation (Additional file 1: Table S1).

### Statistical analysis

If not stated otherwise, statistical analyses were performed using R version 3.2.3.

#### Group differences

Group differences between mRNA expression in LCLs of a deletion carrier compared to his non-carrier parents were tested using *t* test. Group differences of morphological parameters were tested using ANOVA and pairwise ANOVA (type III error) with Tukey’s honest significant difference (HSD) correcting for multiple testing; Tukey’s FDRs were considered significant below a threshold of FDR ≤ 0.05. Group differences between treated and untreated samples (e.g., PI assays) were tested using *t* test; uncorrected *p* values are reported. All samples were compared to their respective control (e.g., non-targeting siRNA) or reference (e.g., 0 mM of inhibitor).

#### Correlations

Correlation between morphological parameters and gene expression or eigengene expression was tested based on the Pearson’s product moment correlation coefficient following a t-distribution. Reported *p* values are Bonferroni corrected for 126 tests (6 morphological parameters, 20 gene modules, 1 gene expression).

#### Targeted mRNA expression

Data of real-time RT-PCR experiments were analyzed using the 2^ΔΔCt^ method [55]. Group differences were tested using *t* test. For validation of RNA-Seq data, real-time RT-PCR data and RNA-Seq (MACE) normalized reads were tested for correlation as described above using Pearson’s correlation tests.

#### Transcriptome analysis

Overall 32,739 transcripts were targeted by MACE analysis and defined as the gene universe (reference gene panel). Differentially expressed genes were identified using the “DESeq2” pipeline [56] with the gene expression as the dependent variable and the respective groups as the independent variable. No additional covariates have been included since the samples did not differ with respect to RIN, number of total counts, or batch. Hierarchical cluster analysis using the top 2000 genes based on variance was performed to exclude any technical outlier samples (for detailed quality checks see Additional file 1: Figure S6). Four different cell lines underwent MACE analysis: the untreated wild-type (WT), the empty control vector (eCtrl), the KO cell line *QPRT*^*−/−*^ del268T, and the KO cell line *QPRT*^*−/−*^ ins395A. Three technical replicates were sequenced for each cell line. Differential gene expression induced by *QPRT-KO* was assessed by comparing (i) WT vs eCtrl, (ii) eCtrl vs KO del268T as well as (iii) eCtrl vs KO ins395A independently. A gene was considered to be significantly associated with *QPRT-KO* if (i) no significant change was identified between the WT and the eCtrl cell line (FDR > 0.1), (ii) if a significant (FDR < 0.05) difference was observed between both the eCtrl and KO del268T and between eCtrl and KO ins395A, and (iii) the direction of the effect was the same in both of the comparisons eCtrl vs KO del268T and eCtrl vs KO ins395A, respectively. For the differential expression analysis between two groups, raw counts of the respective replicates were loaded into DESeq2 using the “DESeqDataSetFromMatrix” function. Differential expression was estimated using the function “DESeq” with the option “fitType = ‘local’”. Significantly up and downregulated genes underwent GO term analysis (Additional file 1: Supplementary Methods). Genes surviving quality check (more than 10 reads per gene in at least 6 of the 12 samples) were subjected to weighted gene co-expression network analysis (WGCNA; Additional file 1: Supplementary Methods).

#### Gene list enrichment test in gene networks of brain development

To gain a deeper insight into the brain-specific effects of up and downregulated genes from MACE analysis, we used part of a framework proposed by Yousaf et al. [57]. In short, this framework uses the Allen Brain Atlas dataset of Kang and colleagues, who have identified 29 co-regulated gene sets using the spatiotemporal transcriptome of the human brain from early embryonal development to late adulthood [34]. Each module corresponds to specific biological processes involved in brain development and aging. We tested these modules for enrichment with the sets of genes up or downregulated upon *QPRT-KO* in SH-SY5Y using Fisher’s exact tests. Expression patterns of the enriched modules were then visualized using heatmaps of the eigenvalues of the respective modules for each human brain region over time.

## Results

### Patient-specific lymphoblastoid cell lines (LCLs)

To replicate previous reports [11] of altered *QPRT* expression in 16p11.2 CNV carriers, we compared *QPRT* expression in a patient-specific LCL of a deletion carrier and his unaffected parents. We confirmed a dosage dependent expression of *QPRT* at mRNA level (16p11.2 deletion carrier vs. non-carrier parents; logFC = − 0.68, *p* = 0.014; Additional file 1: Figure S1a).

### Correlation of QPRT and neuritic complexity in SH-SY5Y wild-type cells

RNA expression of *QPRT* during SH-SY5Y differentiation significantly correlated between microarray [28] and real-time RT-PCR (*ρ* = 0.88; *p* = 0.0098; Additional file 1: Figure S1b). Further, we used the expression data of *QPRT* as well as of the modules of genes co-regulated during neuronal differentiation and tested them for their correlation with morphological parameters using Sholl analysis during 11 days of neuronal differentiation (Additional file 1: Figure S1c and d). Both *QPRT* expression and the eigenvalue of its associated module (MEorange) significantly correlated with the average number of intersections (*QPRT* microarray: *ρ* = 0.86, FDR = 8.16E−05; *QPRT* RT-PCR: *ρ* = 0.54, FDR = 0.020; MEorange: *ρ* = 0.93, FDR = 1.71E−07; Additional file 1: Figure S1d).

In summary, we report a correlative association between *QPRT* expression and a measure for the development of neurite complexity during in vitro neuronal differentiation of wild-type SH-SY5Y cells. To investigate if the correlation of *QPRT* expression with neuritic complexity is causal or secondary, i.e., due to the progressive neurite growth over time, we performed functional inhibition analysis of *QPRT*.

### Functional validation in neuroblastoma cell line SH-SY5Y

#### siRNA mediated knockdown (KD) of QPRT

**Fig. 1:**
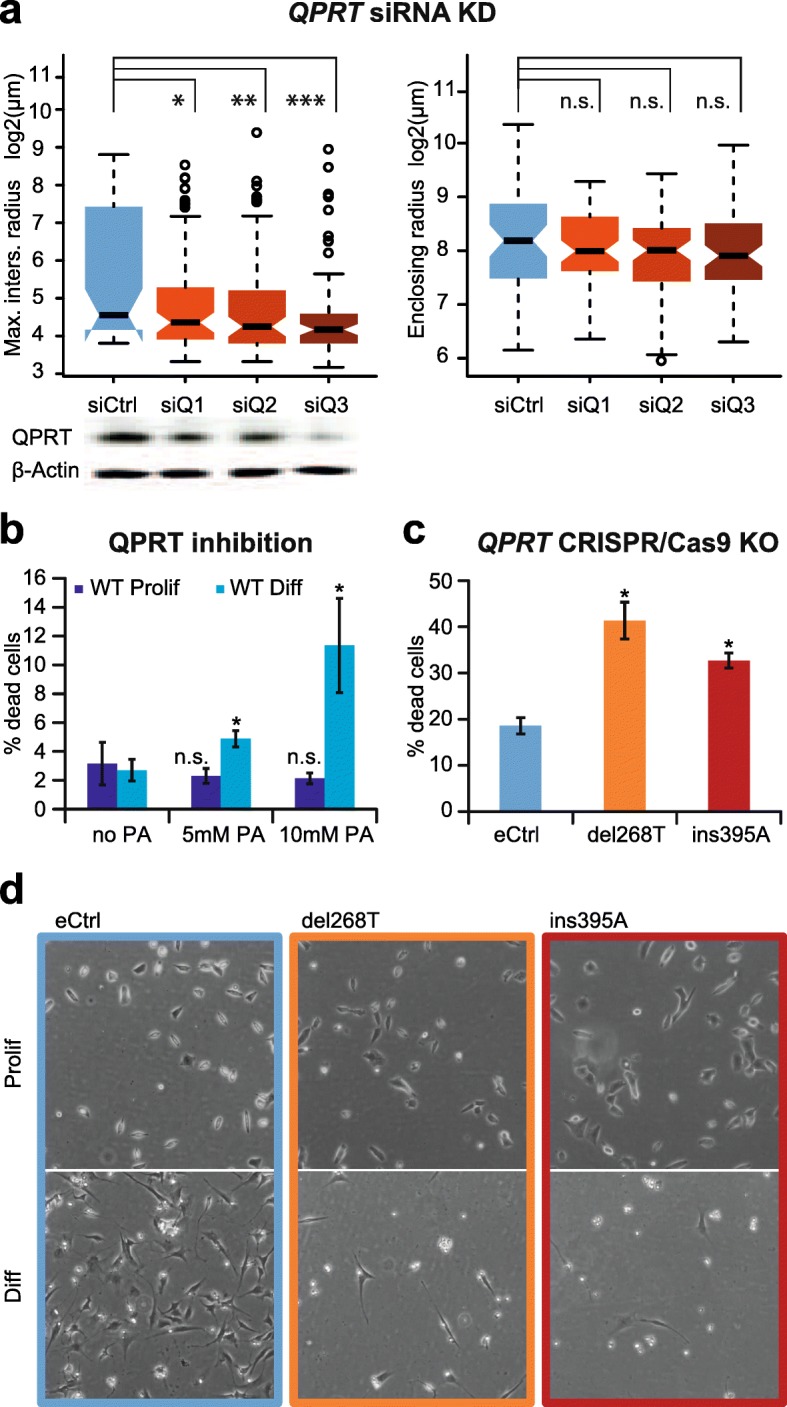
Functional analysis of *QPRT* in SH-SY5Y cells. **a** siRNA-mediated knockdown (KD) of *QPRT*. Cells were transfected with a non-targeting siRNA (siCtrl) and three different siRNAs targeting *QPRT* (siQ1–siQ3). Transfected cells were differentiated for 11 days followed by morphological analysis. Knockdown of QPRT was confirmed at protein level. Upon *QPRT-KD*, cells showed a significant decrease of the maximum intersections radius when compared to the non-targeting control, i.e., the maximum complexity of neurites was significantly closer to the cell soma. None of the three KDs differed with respect to the enclosing radius when compared to the non-target control, i.e., the length of the neurites was not different (Additional file 1: Figure S2). Maximum intersections radius: radial distance of the maximum number of intersections from the cell body. Enclosing radius: outer radius intersecting the cell. All *p* values were corrected for multiple testing using Tukey's HSD correction. **b** Chemical inhibition of *QPRT*. Application of the QPRT inhibitor phthalic acid (PA) for 3 days led to a dose-dependent significant increase of cell death in differentiating wild-type SH-SY5Y cells. In proliferating cells, QPRT inhibition did not change the rate of cell death. **c** Viability assays of CRISPR/Cas9 mediated *QPRT-knock out* (KO) cells. Percentage of cell death was assessed performing viability assays after 3 days of differentiation showing a significant increase of cell death in both generated *QPRT-KO* cell lines. **d** Representative images of *QPRT-KO* cells after differentiation. KO of *QPRT* led to observable cell death during 9 days of differentiation but not during proliferation

All three siRNAs targeting three different sites of *QPRT* (named here siQ1–siQ3) induced a decrease in QPRT protein after 11 days of differentiation as confirmed by Western Blot (Fig. 1a). At neuromorphological level, KD cell lines compared to control cell lines (siCtrl) showed a significant decrease of the maximum intersections radius, altering neuritic complexity in that the site of highest branching density was shifted closer towards the soma (*p* siQ1 0.027, siQ2 0.001, siQ3 3.8E−04; means [SD] siCtrl 103.98[115.54]; siQ1 56.50[80.53]; siQ2 56.29[97.17]; siQ3 41.51[76.70]). The overall cell size or the enclosing radius, did not change upon KD of *QPRT* (all *p* > 0.6; means [SD] siCtrl 349.60[223.68]; siQ1 286.98[125.05]; siQ2 270.06[135.62]; siQ3 293.81[176.86]; Fig. 1a; Additional file 1: Figure S2).

#### Mimicking of QPRT loss

Cells exposed to an inhibitor of QPRT, the chemical phthalic acid (PA), showed an increased cell death upon induction of differentiation, and thus no end-point morphological analysis was performed. Application of PA for 3 days led to a dosage-dependent increase of cell death during differentiation (5 mM PA: FC = 1.81, *p* = 0.019; 10 mM PA: FC = 4.21, *p* = 0.039) but not during proliferation (5 mM PA: FC = 0.73, *p* = 0.432; 10 mM PA: FC = 0.68, *p* = 0.355; Fig. 1b).

We further hypothesized that inhibition of *QPRT* might lead to increased levels of its neurotoxic substrate quinolinic acid (QUIN). Thus, we exposed wild-type cells to elevated QUIN levels. However, we did neither observe an increase in cell death during proliferation (all *p* > 0.3 for 5 μM and 250 μM QUIN) nor during differentiation (all *p* > 0.3 for 5 μM and 250 μM QUIN; Additional file 1: Figure S5a) compared to vehicle only (0 μM). Since the findings from chemical inhibition and mimicking of QPRT inhibition suggested that *QPRT* is causally linked to differentiation deficits independent of its substrate QUIN, we aimed at elucidating the underlying processes in SH-SY5Y cells with a stable loss of *QPRT*.

#### CRISPR/Cas9 mediated knock out (KO) of QPRT

##### Generation and viability

Using two separate sgRNA sequences (see Additional file 1: Supplementary Methods and Supplementary Results), we generated two homozygous *QPRT-KO* cell lines, del268T and ins395A (NM_014298), both of which showed a nearly complete loss of expression of *QPRT* mRNA expression (proliferating cell lines: del268T: below detection limits, ins395A: FC = 0.17, *p* = 0.046; 2 days of differentiation: del268T: FC = 0.13, *p* = 0.003; ins395A: FC = 0.15, *p* = 0.003; all compared to the proliferating empty control vector (eCtrl); Additional file 1: Figures S3 and S4)*.* For proliferating cells, QPRT-KO was furthermore descriptively confirmed at protein level (Additional file 1: Figure S4). While the eCtrl and both *QPRT-KO* cell lines were growing comparably during proliferation, both KO cell lines died upon differentiation. After 3 days of differentiation, we observed a significant increase of cell death in the KO cell lines when compared to eCtrl cells harboring the empty control vector (eCtrl to del268T: FC = 2.23, *p* = 8.2E−04; eCtrl to ins395A: FC = 1.76, *p* = 5.2E−04; Fig. 1c), and after 9 days of differentiation, merely no differentiating KO cells were detected under the microscope (Fig. 1d).

##### Rescue experiments using small compounds

We tried to rescue the *QPRT-KO* effect by inhibiting the effector pathways downstream of QUIN, i.e., inhibit a potentially induced neurotoxicity by hyperactivation of NMDA-R as well as replenish NAD^+^, which is the downstream metabolite of QPRT as well as the final outcome of the kynurenine pathway. Neither the NMDA-R antagonist MK801 (both *QPRT-KO* compared to eCtrl: FC > 1.7, *p* < 3.9E−03 for 0 μM; FC > 1.6, *p* < 0.04 for 6 μM; FC > 1.6, *p* < 1.5E−03 for 12 μM) nor the inhibition of the NMDA-R downstream enzyme NOS1 by L-NAME (both *QPRT-KO* compared to eCtrl: FC > 1.3, *p* < 0.05 for 0 mM; FC > 1.3, *p* < 0.02 for 0.5 mM; FC > 1.5, *p* < 0.01 for 1 mM) nor supplying NAD^+^, the downstream product of tryptophan catabolism (both *QPRT-KO* compared to eCtrl: FC > 1.3, *p* < 9E−03 for 0 mM; FC > 1.3, *p* < 1.6E−03 for 5 mM; del268T compared to eCtrl: FC = 1.20, *p* = 0.1; ins395A compared to eCtrl: FC = 1.54, *p* = 0.009 for 10 mM), resulted in a significant reduction of cell death for both of the KO cell lines upon differentiation (Additional file 1: Figure S5b).

##### Metabolite analysis

To test if the loss of *QPRT* results in an altered metabolite profile of the kynurenine pathway, we implemented UHPLC and GC/MS analysis. We were able to detect tryptophan (TRP), kynurenine (KYN), kynurenic acid (KA), 3-hydroxykynurenine (3HK), anthranilic acid (AA), and picolinic acid (PA). QUIN could not be detected in any of the samples, while 3-hydroxyanthranilic acid (3HAA) could only be detected in the differentiated (eCtrl and both of the KO) cell lines only. However, no significant changes could be observed in any of the metabolites when comparing both of the *QPRT-KO* cell lines to the control cell lines (Additional file 1: Figure S5c).

##### MACE transcriptome analysis of *QPRT-KO*

**Fig. 2:**
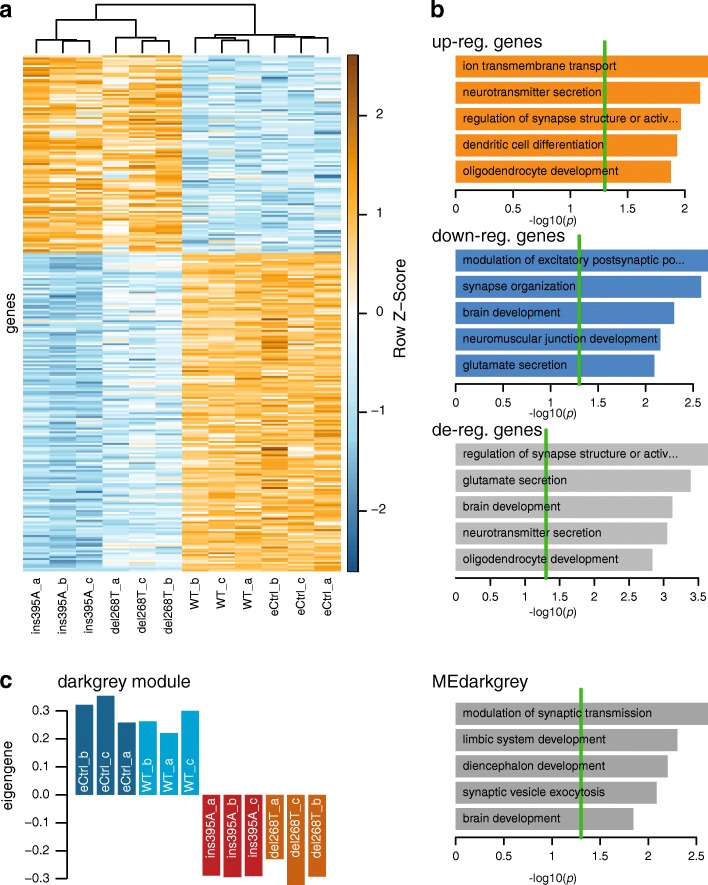
Whole transcriptome analysis of *QPRT-KO* cells and control cell lines. **a** Heatmap of differentially expressed genes. Upon KO of *QPRT,* 269 genes were significantly differentially expressed between both of the KO and the eCtrl cell line (FDR ≤ 0.05) but not between the controls (wild-type and eCtrl, FDR > 0.1). Overall, ins395A descriptively shows stronger effects than del268T. **b** GO term enrichment for differentially regulated genes. Upregulated genes were associated with GO terms including neurotransmitter secretion, negative regulation of cell growth and negative regulation of cytoskeleton organization (all *p* < 0.05; Additional file 3: Table S3). Genes downregulated upon *QPRT-KO* were enriched for GO terms involved in processes of neuronal development (positive regulation of neuron differentiation, positive regulation of dendritic spine development, and synapse organization (all *p* < 0.04)) and neurotransmitter transport (potassium transport, as well as glutamatergic processes like glutamate secretion and regulation of glutamate receptor signaling pathway (all *p* < 0.05)). Deregulated genes were enriched for processes like neurotransmitter secretion and brain development (all *p* < 0.05; Additional file 3: Table S3). All *p* values account for the hierarchical structure of the gene ontology (GO) and can thus be considered as corrected for multiple testing. **c** Regulation of the dark grey gene set. This module was identified as *QPRT-KO* associated module harboring genes downregulated upon *QPRT-KO* when comparing both KO cell lines to wild-type and eCtrl cells (all *p* < 8E−07). GO term enrichment analysis of this module again revealed processes involved in brain development and synaptic transmission and plasticity (*p* < 0.05), confirming the association of *QPRT-KO* with neuronal development

Since in our cell model the effect of *QPRT-KO* on neuronal differentiation was not related to changes in the kynurenine pathway or the neurotoxic effects of QUIN, we aimed to further elucidate the effect of the KO on differentiating cells implementing a transcriptome-wide analysis. Overall, we were able to measure the expression of 32,739 transcripts (Additional file 2: Table S2). Statistical analysis identified 269 differentially regulated genes (103 upregulated and 166 downregulated; Fig. 2a; Additional file 2: Table S2) expressed in all three replicates of both KO cell lines, all with an FDR ≤ 0.05. The 12 genes (Table 1) with an absolute log2FC > 2.5 were technically validated using real-time RT-PCR with an average correlation between RNA sequencing data and real-time RT-PCR of *ρ* = 0.91 (SD = 0.10). *QPRT-KO* was also confirmed (eCtrl vs del268T: log2FC = − 2.44, FDR = 1.07E−207; eCtrl vs ins395A: log2FC = − 3.17, FDR = 9.88E−291). Of the genes coding for components of the kynurenine pathway, only *NMNAT2* was significantly downregulated upon *QPRT-KO* (eCtrl vs del268T: log2FC = − 0.70, FDR = 2.10E−07; eCtrl vs ins395A: log2FC = − 1.51, FDR = 6.70E−28). The ASD-implicated protein NLGN3 was differentially downregulated in the del268T only (eCtrl vs del268T: log2FC = − 0.43, FDR = 6.58E−02; eCtrl vs ins395A log2FC = − 0.09, FDR = 9.15E−01) and was thus not considered to be regulated upon KO of *QPRT*.

**Table 1:** Differentially expressed genes upon *QPRT-KO* with |log2FC| > 2.5

Gene	Chr	del268T vs eCtrl		ins395A vs eCtrl		WT vs eCtrl		SFARI score	ASD literature
		FDR	log2FC	FDR	log2FC	FDR	log2FC		
*COX17*	3q13.33	3.33E−138	− 5.94	1.78E−154	− 5.63	0.30	− 0.13	/	/
*GUCA1A*	6p21.1	4.56E−41	− 5.38	3.97E−46	− 5.88	0.20	0.26	/	/
*COX17P1*	13q14.13	1.09E−84	− 5.19	5.82E−93	− 5.28	0.59	− 0.11	/	/
*VSTM2A*	7p11.2	1.81E−10	− 5.00	6.36E−12	− 5.28	0.61	− 0.27	/	/
*KCNQ3*	8q24.22	5.77E−04	− 4.76	1.25E03	− 3.04	0.46	0.49	3	Role for KCNQ3 in epilepsy and autism [75]
*CCK*	3p22.1	2.56E−04	− 3.18	5.97E−05	− 3.13	0.17	0.68	/	Candidate gene for Asperger’s in a microdeletion case study [76]
*GABRB3*	15q12	3.04E−43	− 3.00	9.43E−54	− 3.58	0.80	0.04	2	CNV Chr15q11-13 implicated in ASD; polymorphisms associated with ASD [62, 77]
*BRINP1*	9q33.1	8.93E−09	− 2.92	2.09E−09	− 2.85	0.20	− 0.53	/	−/− mice: autism-like behavior including reduced sociability and altered vocalization [78]
*LINC01760*	1p21.3	1.02E−02	2.74	4.93E−03	2.62	0.95	− 0.30	/	/
*SNTG2*	2p25.3	7.04E−04	− 2.71	5.06E−07	− 5.58	0.72	0.22	4	Region linked with ID [64], associated with ASD [65]; interaction partner of neuroligins, interaction altered by ASD associated mutations [66]
*ARHGAP20*	11q23.1	1.63E−09	− 2.65	4.08E−15	− 3.93	0.45	− 0.30	/	/
*SRRM4*	12q24.23	1.29E−18	− 2.61	2.25E−26	− 3.40	0.54	− 0.20	/	−/+ mice: multiple autistic-like features [79]

*Chr* chromosomal region*, del268T* CRISPR/Cas9 induced mutation (deletion of one nucleotide) in exon 2 of QPRT, *ins395A* CRISPR/Cas9-induced mutation (insertion of one nucleotide) in exon 2 of QPRT, *eCtrl* control cell line with empty CRISPR/Cas9 control vector, *WT* wild-type SH-SY5Y cell line untreated, *FDR* False discovery rate, *log2 FC* log2 fold change, *SFARI score* score in the SFARI database [58] (a smaller score means higher evidence), *ASD references* Pubmed was searched for “gene and autism” and “gene and ASD”

The genes upregulated upon KO of *QPRT* were enriched (all *p* values < 0.05, Fig. 2b; Additional file 3: Table S3) for GO annotated biological processes involved in neurotransmitter secretion, regulation of synapse structure, or activation (Fig. 2b) but also negative regulation of cell growth and negative regulation of cytoskeleton organization among others (Additional file 3: Table S3). In addition, upregulated genes were enriched for the GO term apoptotic process involved in morphogenesis via the genes *BCL2 antagonist/killer 1* (*BAK1*) and *scribbled planar cell polarity protein* (*SCRIB*). Genes downregulated in KO cell lines showed enrichment for GO terms including synapse organization, modulation of excitatory postsynaptic potentials, or glutamate secretion (Fig. 2b) or positive regulation of neuron differentiation, positive regulation of dendritic spine development as well as ion transmembrane transport (Fig. 2b; Additional file 3: Table S3). We also observed significant enrichment for ASD genes among highly differentially regulated candidates. Of the 269 differentially regulated genes, 15 were listed in the SFARI gene [58] with an evidence score of 4 or better (*p* = 9.2E−04, odds ratio OR [95% confidence interval] = 2.68 [1.47–4.54]), for example *gamma-aminobutyric acid type A receptor beta 3 subunit* (*GABRB3*), *potassium voltage-gated channel subfamily Q member 3* (*KCNQ3*), *syntrophin gamma 2* (*SNTG2*), or *contactin associated protein-like 2* (*CNTNAP2*).

A total of eleven out of 166 downregulated genes and one gene of 103 upregulated genes showed a |log2FC| > 2.5 (Table 1). Literature search for these twelve highly regulated genes revealed that six had been published in the context of ASD before (Table 1).

##### Gene network analysis

At gene network level, we identified 20 co-regulated modules upon KO. Here, *QPRT* was co-regulated within a module associated with modulation of synaptic transmission, synaptic vesicle exocytosis, or limbic system and diencephalon development (Fig. 2c) as well as neurotransmitter secretion or negative regulation of neurogenesis (*p* < 0.04). Overall, the eigenvalue of this module was not different between controls and KOs. We identified one co-regulated gene set (dark grey, Fig. 2c) to be associated with *QPRT-KO* as it was significantly downregulated in both KO cell lines when compared to the control cell lines (KOs versus eCtrl or WT, all *p* < 8E−07). GO term enrichment analysis of this module again revealed processes involved in brain development and synaptic transmission and plasticity (*p* < 0.05; Fig. 2c; Additional file 3: Table S3), confirming the association of *QPRT-KO* with neuronal development.

##### Translation to developmental brain expression data

**Fig. 3:**
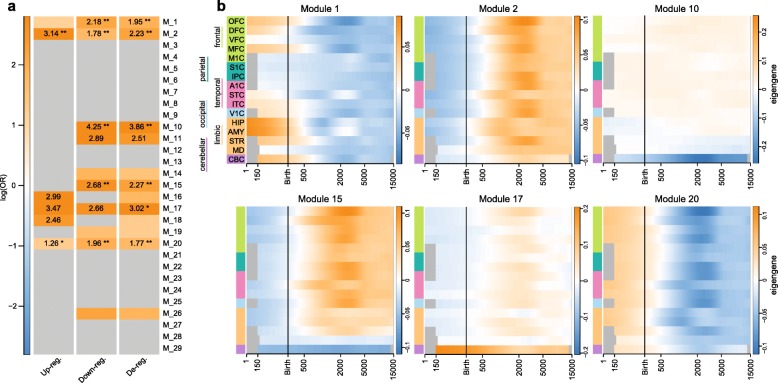
Translation of *QPRT-KO*-induced gene expression profile to human brain development. **a** Modules enriched for *QPRT-KO* de-regulated genes. A total of 29 modules (in the following termed “Kang-Modules”) identified by Kang and colleagues [34] were tested for enrichment with genes differentially regulated in *QPRT-KO* cells. Downregulated genes were strongly enriched in the Kang-Module number 1, 10, 15, and 20 while upregulated genes were enriched in Kang-Module 2 number and 20. Kang-Module number 17 was enriched for deregulated genes in general, i.e., for the merged lists of up and downregulated genes. **b** Regulation of modules enriched for *QPRT-KO* implicated genes during brain development. Kang-Modules number 15 (enriched for *QPRT*-KO induced downregulated genes), 2, and 20 (both enriched for genes up and downregulated) are strongly regulated during early infancy (~ 2 years), especially in the dorsolateral prefrontal cortex, the superior temporal cortex, hippocampus, and amygdala. Kang-Module number 10 is downregulated in the cerebellum while it is enriched for genes downregulated in *QPRT-KO* cells. Kang-Module number 17 is enriched for deregulated genes and shows an upregulation of genes expressed in the cerebellum. Kang-Module number 1 shows strong regulation of hippocampus and amygdala, parts of the frontal cortex, and the cerebellum. This module is enriched for genes downregulated in *QPRT-KO* cells. *Abbreviations*: OFC orbital prefrontal cortex, DFC dorsolateral prefrontal cortex, VFC ventrolateral prefrontal cortex, MFC medial prefrontal cortex, M1C primary motor (M1) cortex, S1C primary somatosensory (S1) cortex, IPC posterior inferior parietal cortex, A1C primary auditory (A1) cortex, STC superior temporal cortex, ITC inferior temporal cortex, V1C primary visual (V1) cortex, HIP hippocampus, AMY amygdala, STR striatum, MD mediodorsal nucleus of the thalamus, CBC cerebellar cortex

Finally, we aimed to translate the effects of *QPRT-KO* onto the spatial and temporal gene expression network of the brain using previously published data [34]. In this previous work, the authors report 29 gene modules (termed “Kang-Module” in the following) that are co-regulated during the development of human brain regions. We observe that the *QPRT-KO*-induced downregulated genes were strongly enriched in the Kang-Modules number 1 (odds ratio OR [95% confidence interval] = 8.86 [3.12–20.45], *p* = 5.76E−04), number 2 (OR = 5.96 [3.55–9.75], *p* = 2.21E09), number 10 (OR = 69.92 [20.70–188.44], *p* = 2.64E−07), number 15 (OR = 14.58 [6.00–30.72], *p* = 1.57E−06) and number 20 (OR = 7.13 [4.40–11.40], *p* = 7.74E−13) while the *QPRT-KO* upregulated genes were enriched in Kang-Modules number 2 (OR = 23.20 [11.25–49.97], *p* = 1.07E−16) and number 20 (OR = 3.54 [1.46–7.76], *p* = 4.18E−02). Kang-Module 17 was enriched for *QPRT*-*KO*-induced deregulated genes in general, i.e., for the merged lists of up and downregulated genes (OR = 20.41 [2.33–82.57], *p* = 2.45E−02; Fig. 3a). By plotting the eigengene value of the Kang-Module over time for each region, we observed that number 1 shows strong early upregulation in the hippocampus (HIP) and the amygdala (AMY) during embryonal development while it is downregulated in all tested brain regions after birth (Fig. 3b). Kang-Module 1 is also slightly upregulated in embryonal development in parts of the frontal cortex (orbitofrontal cortex (OFC), dorsolateral prefrontal cortex (DFC) and medial prefrontal cortex (MFC)), the striatum (STR), and the cerebellar cortex (CBC). Kang-Module 2 is overall downregulated prenatally and shows upregulation after birth, with maximum expression between the ages of 6 and 14, i.e., during middle and late childhood in OFC, MFC, posterior inferior parietal cortex (IPC), primary auditory (A1) cortex (A1C), inferior temporal cortex (ITC), primary visual (V1) cortex (V1C), and STR. Kang-Module 10 is only expressed in the CBC with a peak around the age of 6 years, while Kang-Module 15 is strongly downregulated during embryonal development in the CBC. The low expression of Kang-Module 15 in the CBC is stable across the tested time course, and it is slightly upregulated in other brain regions, showing peaks between the years 6 and 14 in OFC, DFC, ventrolateral prefrontal cortex (VFC), MFC, IPC, A1C, ITC, and V1C. Kang-Module 17 shows a constant active expression in the CBC over time with a peak during early development in the CBC. Kang-Module 20 is an overall early upregulated gene network downregulated after the age of ~ 2 years, showing the strongest downregulation between the years 6 and 14 in OFC, MFC, IPC, A1C, ITC, V1C, and STR.

## Discussion

Based on our differential analysis, we report a causal relation between *QPRT*, located in the ASD-associated CNV region Chr16p11.2 and neuronal differentiation of SH-SY5Y cells. A gene dosage reduction or inhibition of QPRT affects morphological parameters during neuronal differentiation as well as the regulation of genes and gene networks that were previously implicated in ASD. This includes processes like synapse organization or brain development. In summary, our findings suggest a neurodevelopmental role for *QPRT* in the etiology of ASD in 16p11.2 deletion carriers.

As expected from previous findings [11], in one deletion carrier with ASD, we confirmed that *QPRT* was expressed in a gene dosage-dependent manner strengthening the role of *QPRT* as a potential risk gene in the pathology of Chr16p11.2 deletion syndrome. By comparing our previously published transcriptome data of SH-SY5Y wild-type cells [28] to the morphological changes during neuronal differentiation described in the present study, we found *QPRT* to be correlated with the development of the neuritic complexity. These results suggest a potential regulatory link between *QPRT* and neuronal maturation. Our findings of the KD and KO cell models further underline this interpretation: the KD of *QPRT* led to subtle changes of SH-SY5Y neuronal complexity in line with previous reports using mouse models of Chr16p11.2 [17] as well as iPS cells generated from 16p11.2 CNV carriers [59] and postmortem studies of ASD individuals [1]. Inhibition of QPRT activity as well as genetic KO both led to cell death of differentiating but not proliferating cells. These findings suggest that enough protein is left for the survival of differentiating cells upon KD of QPRT while the loss of QPRT is lethal for differentiating SH-SY5Y cells. Interestingly, the administration of the QPRT substrate QUIN, which is a potent excitotoxin, did not show any effect on neither proliferating nor differentiating cells. Previous findings suggested that a reduction of QPRT, the only enzyme catabolizing quinolinic acid (QUIN), leads to an accumulation of QUIN, which in turn may induce neuronal cell death by over-activating NMDA-R and increase nitric oxide (NO) production [46]. Altered QUIN levels could also lead to a change in NAD^+^ production which in turn could change poly (ADP-ribose) polymerase (PARP) activity [60]. In *QPRT-KO* mice, the striatum showed an accumulation of QUIN leading to neurodegeneration [30] as well as altered expression of enzymes of the kynurenine pathway and of NMDA-receptors. No ASD-like behaviors were studied in these mice, but the animals showed no growth or developmental abnormalities. As the kynurenine pathway was associated with Parkinson’s disease [61], the authors performed a behavioral test measuring the stride length of WT and KO mice. Indeed, they reported shorter stride lengths in aged but not middle-aged *QPRT-KO* mice as usually seen in mouse models of Parkinson’s disease [30]. In another study, no differences of histological features of the cerebrum of *QPRT-KO* mice were observed [31]. Although we were not able to mimic QPRT loss in WT cells by application of increased QUIN levels, we tried to rescue differentiating QPRT-KO cells from cell death during differentiation by modulation of QUIN-related metabolism and signaling. Inhibition of NMDA-receptors was expected to protect *QPRT-KO* cells from the possible QUIN-induced excitotoxicity. However, the application of different concentrations of an NMDA-R inhibitor did not prevent cell death. Similarly, inhibition of NOS1 with the aim to reduce the production of NO did not affect viability. Finally, we tried to increase the viability of differentiating cells via the application of NAD^+^ to rescue PARP activity. Again, *QPRT-KO* cells could not be rescued from cell death during differentiation. In accordance with these observations, *QPRT-KO* cells did not show changes in the expression levels of any genes coding for PARP enzymes. In addition, QUIN could not be detected in any of our samples, and none of the metabolites of tryptophan catabolism was significantly changed upon KO of *QPRT* in the performed metabolite assays. Taken together, we conclude that the detrimental effect of QPRT loss on viability during SH-SY5Y differentiation is independent of QUIN levels as well as of QUIN-induced metabolic changes or the kynurenine pathway in general.

To elucidate the underlying mechanisms of *QPRT-KO*-induced cell death during SH-SY5Y neuronal differentiation, we performed a hypothesis-free transcriptomic approach. These findings suggest that loss of *QPRT* may lead to an increased negative regulation of cytoskeleton organization and to an inhibition of neuronal differentiation and dendritic spine development. In our KD model, we observed an alteration of neuritic complexity, which strongly supports the functional role of *QPRT* in these processes.

The functional association of *QPRT* with ASD is supported by the following results of our study: *QPRT-KO* led to inhibition of *GABRB3*, which has been well established as an ASD risk gene [62]. *GABRB3* codes for a subunit of an inhibitory GABA receptor. It is located on Chr15q11-13, a region strongly implicated in ASD [63]. Similarly, the gene *SNTG2* also downregulated in *QPRT-KO* cells, codes for a synaptic scaffolding protein involved in actin and PDZ domain binding. *SNTG2* is located in 2p25.3, a region linked to intellectual disability [64] and associated with ASD [65]. Furthermore, SNTG2 protein interacts with *neuroligins* (*NLGN*), and this interaction is altered by ASD associated mutations in the *NLGN* genes [66]. QPRT was also found to physically interact with NLGN3 (neuroligin 3; [33]). Although the function of this interaction is still unclear, it is likely that QPRT is involved in the formation of the postsynaptic density of GABAergic neurons. *KCNQ3*, another ASD candidate gene, downregulated upon *QPRT-KO*, encodes a protein regulating neuronal excitability. Variants of this gene were found to be implicated in the development of ASD and epilepsies [67]. Finally, we also found *CNTNAP2*, a well-replicated risk gene for ASD [68] located in 7q22-q36, to be downregulated in *QPRT-KO* cells. In a previous study, we reported *CNTNAP2* promoter variants reducing transcription to be risk factors for ASD [69].

Besides ASD-associated differentially regulated genes, we report six novel candidates (i.e., *COX17*, *GUCA1A*, *COX17P1*, *VSTM2A*, *LINC01760*, and *ARHGAP20*). At this stage, we cannot find any link to neuronal development for the photoreceptor-associated g*uanylate cyclase activator 1A GUCA1A*, the preadipocyte development implicated *V-set and transmembrane domain containing 2A VSTM2A* gene, and the long intergenic non-coding RNA *LINC01760*. However, the *cytochrome c oxidase copper chaperone COX17* (and its pseudogene *COX17P1*) and the *Rho GTPase-activating protein 20 ARHGAP20* are functionally related to neuronal development: COX17 is part of the terminal component of the mitochondrial respiratory chain, catalyzing the electron transfer from reduced cytochrome c to oxygen, and might thus be involved in the regulation of oxidative stress and energy metabolism, both processes that have previously been associated with ASD [36]. The ARHGAP20 enzyme is implicated in neurite outgrowth and thus potentially associated with the here observed neuromorphological phenotypes [70].

Translating the in vitro transcriptomic effect of *QPRT-KO* in SH-SY5Y cells to the gene networks active during human brain development [34], we observed differentially regulated genes to be enriched in modules previously associated with cell cycle regulation (Kang-Module 1), transcription factors regulating progenitor cell fate (Kang Module 20), neuronal development (Kang-Module 10 and 20), morphogenesis (Kang Modules 17 and 2), and synaptic transmission (Kang-Module 2 and 15). In addition, the Kang-Modules identified to be affected by *QPRT-KO* were also reported to be highly co-expressed (*ρ* ≥ 0.68) with markers for glutamatergic (Kang-Module 10) and GABAergic neurons (Kang-Modules 2 and 15) or astrocytes (Kang-Module 2). In addition, Kang-Module 1 is implicated in the early development of the hippocampus and amygdala, two regions significantly associated with ASD after meta-analysis [71, 72]. These associations identified in the translational approach suggest that *QPRT* loss might trigger alterations in the development of excitatory/inhibitory neuronal networks, a pathomechanism postulated to underlie the etiology of ASD [73, 74].

In summary, our findings allow us to conclude that in the neuroblastoma model SH-SY5Y a loss of *QPRT* impairs neuronal development in vitro by changing genetic networks previously implicated in the etiology of ASD. To confirm these results and the role of *QPRT* in the etiology of ASD in general, further studies in other neuronal or animal models are needed. In particular, analyzing the above described *QPRT-KO* mouse model [30, 31] could elucidate the effect of QPRT loss at systems level.

## Conclusions

Here, we report *QPRT*, a gene within the ASD-associated 16p11.2 CNV region, to be essential for and causally related to SH-SY5Y neuronal differentiation in vitro*.* This corroborates postmortem findings of a disturbed neuronal development in ASD. The functional mechanism is still elusive; however, based on our results, we can exclude alterations of QUIN levels or other products of the kynurenine pathway in the here described SH-SY5Y model. Further, the transcriptomic approach suggests that a reduced availability of *QPRT* impacts on genes and networks associated with ASD, such as neuron differentiation, synapse organization, or the development of excitatory/inhibitory neurons. Overall this study suggests an alteration of *QPRT* expression or of *QPRT* related genes to be underlying the etiology of ASD in 16p11.2 CNV carriers. Further studies are needed to confirm our findings in more mature neuronal systems or animal models.

## Additional files

Additional file 1: Supplementary information: Supplementary methods, supplementary results, supplementary table S1, supplementary figures S1-S6. (DOCX 555 kb)

Additional file 2: Supplementary table S2. Results from DESeq2 analysis and count data from RNA-Seq (MACE analysis). (XLSX 5308 kb)

Additional file 3: Supplementary table S3. Results from GO term enrichment analysis. (XLSX 36 kb)

## Abbreviations

3HAA: 3-Hydroxyanthranilic acid
3HK: 3-Hydroxykynurenine
A1: Primary auditory
A1C: Primary auditory (A1) cortex
AA: Anthranilic acid
ACTB: β-Actin
ADI-R: Autism Diagnostic Interview-Revised
ADORA2A: Adenosine receptor 2A
ADOS: Autism Observation Schedule
AMY: Amygdala
ARHGAP20: Rho GTPase-activating protein 20
ASD: Autism spectrum disorder
BAK1: BCL2-antagonist/killer 1
BDNF: Brain-derived neurotrophic factor
BRINP1: BMP/retinoic acid-inducible neural specific 1
CBC: Cerebellar cortex
CCK: Cholecystokinin
CNTNAP2: Contactin-associated protein-like 2
CNV: Copy number variation
COX17: COX17, cytochrome c oxidase copper chaperone
COX17P1: COX17, cytochrome c oxidase copper chaperone pseudogene 1
del268T: QPRT NM_014298 del268T; Ex2.1
DFC: Dorsolateral prefrontal cortex
DMEM: Dulbecco’s modified Eagle medium
DOC2A: Double C2 domain alpha
DPBS: Dulbecco’s phosphate buffered saline
DRD2: Dopamine receptor D2
eCtrl: Empty control vector
ERK/MAPK: Extracellular signaling-related kinase
FAM57BA: Family with sequence similarity 57, member Ba
FBS: Fetal bovine serum
FC: Fold change
GABRB3: Gamma-aminobutyric acid type A receptor beta3 subunit
GAPDH: Glyceraldehyde-3-phosphate dehydrogenase
GC/MS: Gas chromatography-mass spectrometry
GUCA1A: Guanylate cyclase activator 1A
GUSB: Glucuronidase beta
HIP: Hippocampus
HSD: Honest significance difference
ins395A: QPRT NM_014298 ins395A; Ex2.2
IPC: Posterior inferior parietal cortex
ITC: Inferior temporal cortex
KA: Kynurenic acid
KCNQ3: Potassium voltage-gated channel subfamily Q member 3
KCTD13: Potassium channel tetramerization domain containing 13
KD: Knockdown
KIF22: Kinesin family member 22
KO: Knock out
KYN: Kynurenine
LCL: Lymphoblastoid cell line
LINC01760: Long intergenic non-protein coding RNA 1760
MACE: Massive analysis of cDNA ends
MAPK3: Mitogen-activated protein kinase 3
MFC: Medial prefrontal cortex
NAD: Nicotinamide adenine dinucleotide
NLGN: Neuroligins
NLGN3: Neuroligin 3
NMDA-R: N-methyl-D-aspartate receptor
NMNAT2: Nicotinamide nucleotide adenylyltransferase 2
NO: Nitric oxide
NOS1: Nitric oxide synthase 1
OFC: Orbitofrontal cortex
PA: Phthalic acid
PARP: Poly (ADP-ribose) polymerase
PI: Propidium iodide
PIC: Picolinic acid
PSMD7: Proteasome 26S subunit, non-ATPase 7
QPRT: Quinolinate phosphoribosyltransferase
QUIN: Quinolinic acid
RA: Retinoic acid
RIN: RNA integrity number
SCRIB: Scribbled planar cell polarity protein
siCtrl: Non-targeting siRNA control
siQ1-siQ3: QPRT targeting siRNA1-3
SNTG2: Syntrophin gamma 2
SRRM4: Serine/arginine repetitive matrix 4
STR: Striatum
TAOK2: TAO kinase 2
TBX6: T-box 6
TRP: Tryptophan
UHPLC: Ultra-high-performance liquid chromatography
UPL: Universal probe library
V1: Primary visual
V1C: Primary visual (V1) cortex
VFC: Ventrolateral prefrontal cortex
VSTM2A: V-set and transmembrane domain containing 2A
WGCNA: Weighted gene co-expression network analysis
WT: Wild type

## References

[1] Raymond GV, Bauman ML, Kemper TL (1996). Hippocampus in autism: a Golgi analysis. Acta Neuropathol.

[2] Jacot-Descombes S, Uppal N, Wicinski B, Santos M, Schmeidler J, Giannakopoulos P (2012). Decreased pyramidal neuron size in Brodmann areas 44 and 45 in patients with autism. Acta Neuropathol.

[3] Casanova MF, Kooten v, Imke AJ, Switala AE, van Engeland H, Heinsen H, Steinbusch HWM (2006). Minicolumnar abnormalities in autism. Acta Neuropathol.

[4] Stoner R, Chow ML, Boyle MP, Sunkin SM, Mouton PR, Roy S (2014). Patches of disorganization in the neocortex of children with autism. N Engl J Med.

[5] Sanders SJ, He X, Willsey AJ, Ercan-Sencicek AG, Samocha KE, Cicek AE (2015). Insights into autism spectrum disorder genomic architecture and biology from 71 risk loci. Neuron.

[6] Pinto D, Delaby E, Merico D, Barbosa M, Merikangas A, Klei L (2014). Convergence of genes and cellular pathways dysregulated in autism spectrum disorders. Am J Hum Genet.

[7] Woodbury-Smith Marc, Scherer Stephen W (2018). Progress in the genetics of autism spectrum disorder. Developmental Medicine & Child Neurology.

[8] Stein JL (2015). Copy number variation and brain structure: lessons learned from chromosome 16p11.2. Genome Med.

[9] NIH Genetics Home Reference. https://ghr.nlm.nih.gov/condition/16p112-deletion-syndrome. Accessed 30 Apr 2018.

[10] NIH Genetics Home Reference. https://ghr.nlm.nih.gov/condition/16p112-duplication. Accessed 30 Apr 2018.

[11] Blumenthal I, Ragavendran A, Erdin S, Klei L, Sugathan A, Guide JR (2014). Transcriptional consequences of 16p11.2 deletion and duplication in mouse cortex and multiplex autism families. Am J Hum Genet.

[12] Blaker-Lee A, Gupta S, McCammon JM, de RG, Sive H (2012). Zebrafish homologs of genes within 16p11.2, a genomic region associated with brain disorders, are active during brain development, and include two deletion dosage sensor genes. Dis Model Mech.

[13] McCammon JM, Blaker-Lee A, Chen X, Sive H (2017). The 16p11.2 homologs fam57ba and doc2a generate certain brain and body phenotypes. Hum Mol Genet.

[14] Arbogast T, Ouagazzal A-M, Chevalier C, Kopanitsa M, Afinowi N, Migliavacca E (2016). Reciprocal effects on neurocognitive and metabolic phenotypes in mouse models of 16p11.2 deletion and duplication syndromes. PLoS Genet.

[15] Pucilowska J, Vithayathil J, Tavares EJ, Kelly C, Karlo JC, Landreth GE (2015). The 16p11.2 deletion mouse model of autism exhibits altered cortical progenitor proliferation and brain cytoarchitecture linked to the ERK MAPK pathway. J Neurosci.

[16] Horev G, Ellegood J, Lerch JP, Son Y-EE, Muthuswamy L, Vogel H (2011). Dosage-dependent phenotypes in models of 16p11.2 lesions found in autism. Proc Natl Acad Sci U S A.

[17] Blizinsky KD, Diaz-Castro B, Forrest MP, Schurmann B, Bach AP, Martin-de-Saavedra MD (2016). Reversal of dendritic phenotypes in 16p11.2 microduplication mouse model neurons by pharmacological targeting of a network hub. Proc Natl Acad Sci U S A.

[18] Grissom N M, McKee S E, Schoch H, Bowman N, Havekes R, O'Brien W T, Mahrt E, Siegel S, Commons K, Portfors C, Nickl-Jockschat T, Reyes T M, Abel T (2017). Male-specific deficits in natural reward learning in a mouse model of neurodevelopmental disorders. Molecular Psychiatry.

[19] Toma C, Hervás A, Balmaña N, Salgado M, Maristany M, Vilella E (2013). Neurotransmitter systems and neurotrophic factors in autism: association study of 37 genes suggests involvement of DDC. World J Biol Psychiatry.

[20] Freitag CM, Agelopoulos K, Huy E, Rothermundt M, Krakowitzky P, Meyer J (2010). Adenosine A(2A) receptor gene (ADORA2A) variants may increase autistic symptoms and anxiety in autism spectrum disorder. Eur Child Adolesc Psychiatry.

[21] Golzio C, Willer J, Talkowski ME, Oh EC, Taniguchi Y, Jacquemont S (2012). KCTD13 is a major driver of mirrored neuroanatomical phenotypes of the 16p11.2 copy number variant. Nature.

[22] Ip Jacque P.K., Nagakura Ikue, Petravicz Jeremy, Li Keji, Wiemer Erik A.C., Sur Mriganka (2018). Major Vault Protein, a Candidate Gene in 16p11.2 Microdeletion Syndrome, Is Required for the Homeostatic Regulation of Visual Cortical Plasticity. The Journal of Neuroscience.

[23] Calderon de Anda F, Rosario AL, Durak O, Tran T, Gräff J, Meletis K (2012). Autism spectrum disorder susceptibility gene TAOK2 affects basal dendrite formation in the neocortex. Nat Neurosci.

[24] Li Z, He X, Feng J (2013). 16p11.2 is required for neuronal polarity. WJNS.

[25] Qureshi AY, Mueller S, Snyder AZ, Mukherjee P, Berman JI, Roberts TPL (2014). Opposing brain differences in 16p11.2 deletion and duplication carriers. J Neurosci.

[26] Subramanian M, Timmerman CK, Schwartz JL, Pham DL, Meffert MK (2015). Characterizing autism spectrum disorders by key biochemical pathways. Front Neurosci.

[27] Lin GN, Corominas R, Lemmens I, Yang X, Tavernier J, Hill DE (2015). Spatiotemporal 16p11.2 protein network implicates cortical late mid-fetal brain development and KCTD13-Cul3-RhoA pathway in psychiatric diseases. Neuron.

[28] Chiocchetti AG, Haslinger D, Stein JL, de La T-UL, Cocchi E, Rothamel T (2016). Transcriptomic signatures of neuronal differentiation and their association with risk genes for autism spectrum and related neuropsychiatric disorders. Transl Psychiatry.

[29] Ting KK, Brew BJ, Guillemin GJ (2009). Effect of quinolinic acid on human astrocytes morphology and functions: implications in Alzheimer's disease. J Neuroinflammation.

[30] Fukuoka S-I, Kawashima R, Asuma R, Shibata K, Fukuwatari T (2012). Quinolinate accumulation in the brains of the quinolinate phosphoribosyltransferase (qprt) knockout mice.

[31] Terakata M, Fukuwatari T, Sano M, Nakao N, Sasaki R, Fukuoka S-I, Shibata K (2012). Establishment of true niacin deficiency in quinolinic acid phosphoribosyltransferase knockout mice. J Nutr.

[32] Lim Chai K., Essa Musthafa M., de Paula Martins Roberta, Lovejoy David B., Bilgin Ayse A., Waly Mostafa I., Al-Farsi Yahya M., Al-Sharbati Marwan, Al-Shaffae Mohammed A., Guillemin Gilles J. (2015). Altered kynurenine pathway metabolism in autism: Implication for immune-induced glutamatergic activity. Autism Research.

[33] Shen C, L-r H, X-l Z, P-r W, Zhong N (2015). Novel interactive partners of neuroligin 3: new aspects for pathogenesis of autism. J Mol Neurosci.

[34] Kang HJ, Kawasawa YI, Cheng F, Zhu Y, Xu X, Li M (2011). Spatio-temporal transcriptome of the human brain. Nature.

[35] Neitzel H (1986). A routine method for the establishment of permanent growing lymphoblastoid cell lines. Hum Genet.

[36] Chiocchetti AG, Haslinger D, Boesch M, Karl T, Wiemann S, Freitag CM (2014). Protein signatures of oxidative stress response in a patient specific cell line model for autism. Mol Autism..

[37] Poustka F, Lisch S, Rühl D, Sacher A, Schmötzer G, Werner K (1996). The standardized diagnosis of autism, autism diagnostic interview-revised: interrater reliability of the German form of the interview. Psychopathology.

[38] Lord C, Rutter M, Le Couteur A (1994). Autism diagnostic interview-revised: a revised version of a diagnostic interview for caregivers of individuals with possible pervasive developmental disorders. J Autism Dev Disord.

[39] Bölte S, Poustka F (2004). Diagnostische Beobachtungsskala für Autistische Störungen (ADOS): Erste Ergebnisse zur Zuverlässigkeit und Gültigkeit. Z Kinder Jugendpsychiatr Psychother.

[40] Schindelin J, Rueden CT, Hiner MC, Eliceiri KW (2015). The ImageJ ecosystem: an open platform for biomedical image analysis. Mol Reprod Dev.

[41] Ristanović D, Milosević NT, Stulić V (2006). Application of modified Sholl analysis to neuronal dendritic arborization of the cat spinal cord. J Neurosci Methods.

[42] ImageJ Sholl Analysis. http://imagej.net/Sholl_Analysis. Accessed 30 Apr 2018.

[43] Ran FA, Hsu PD, Wright J, Agarwala V, Scott DA, Zhang F (2013). Genome engineering using the CRISPR-Cas9 system. Nat Protoc.

[44] CRISPR design. http://crispr.mit.edu/. Accessed 30 Apr 2018.

[45] Braidy N, Guillemin GJ, Grant R (2011). Effects of kynurenine pathway inhibition on NAD metabolism and cell viability in human primary astrocytes and neurons. Int J Tryptophan Res.

[46] Braidy N, Grant R, Adams S, Brew BJ, Guillemin GJ (2009). Mechanism for quinolinic acid cytotoxicity in human astrocytes and neurons. Neurotox Res.

[47] Petroni D, Tsai J, Mondal D, George W (2013). Attenuation of low dose methylmercury and glutamate induced-cytotoxicity and tau phosphorylation by an N-methyl-D-aspartate antagonist in human neuroblastoma (SHSY5Y) cells. Environ Toxicol.

[48] Candemir E, Kollert L, Weissflog L, Geis M, Muller A, Post AM (2016). Interaction of NOS1AP with the NOS-I PDZ domain: implications for schizophrenia-related alterations in dendritic morphology. Eur Neuropsychopharmacol.

[49] Zheng T, Xu SY, Zhou SQ, Lai LY, Li L (2013). Nicotinamide adenine dinucleotide (NAD+) repletion attenuates bupivacaine-induced neurotoxicity. Neurochem Res.

[50] Lim CK, Bilgin A, Lovejoy DB, Tan V, Bustamante S, Taylor BV (2017). Kynurenine pathway metabolomics predicts and provides mechanistic insight into multiple sclerosis progression. Sci Rep.

[51] Zhernakov A, Rotter B, Winter P, Borisov A, Tikhonovich I, Zhukov V (2017). Massive Analysis of cDNA Ends (MACE) for transcript-based marker design in pea (Pisum sativum L.). Genom Data.

[52] Muller S., Rycak L., Afonso-Grunz F., Winter P., Zawada A. M., Damrath E., Scheider J., Schmah J., Koch I., Kahl G., Rotter B. (2014). APADB: a database for alternative polyadenylation and microRNA regulation events. Database.

[53] Nold-Petry CA, Lo CY, Rudloff I, Elgass KD, Li S, Gantier MP (2015). IL-37 requires the receptors IL-18Rα and IL-1R8 (SIGIRR) to carry out its multifaceted anti-inflammatory program upon innate signal transduction. Nat Immunol.

[54] Langmead B, Salzberg SL (2012). Fast gapped-read alignment with Bowtie 2. Nat Methods.

[55] Livak KJ, Schmittgen TD (2001). Analysis of relative gene expression data using real-time quantitative PCR and the 2(-delta delta C(T)) method. Methods.

[56] Love MI, Huber W, Anders S (2014). Moderated estimation of fold change and dispersion for RNA-seq data with DESeq2. Genome Biol.

[57] Yousaf A, Duketis E, Jarczok T, Sachse M, Biscaldi M, Degenhardt F, et al. Mapping the genetics of neuropsychological traits to the molecular network of the human brain using a data integrative approach. bioRxiv. 2018. 10.1101/336776.

[58] SFARI Gene. https://gene.sfari.org/database/human-gene/. Accessed 30 Apr 2018.

[59] Deshpande A, Yadav S, Dao DQ, Wu Z-Y, Hokanson KC, Cahill MK (2017). Cellular phenotypes in human iPSC-derived neurons from a genetic model of autism spectrum disorder. Cell Rep.

[60] Sahm F, Oezen I, Opitz CA, Radlwimmer B, von Deimling A, Ahrendt T (2013). The endogenous tryptophan metabolite and NAD+ precursor quinolinic acid confers resistance of gliomas to oxidative stress. Cancer Res.

[61] Campbell BM, Charych E, Lee AW, Möller T (2014). Kynurenines in CNS disease: regulation by inflammatory cytokines. Front Neurosci.

[62] Buxbaum JD, Silverman JM, Smith CJ, Greenberg DA, Kilifarski M, Reichert J (2002). Association between a GABRB3 polymorphism and autism. Mol Psychiatry.

[63] de La Torre-Ubieta L, Won H, Stein JL, Geschwind DH (2016). Advancing the understanding of autism disease mechanisms through genetics. Nat Med.

[64] Bulayeva K, Lesch K-P, Bulayev O, Walsh C, Glatt S, Gurgenova F (2015). Genomic structural variants are linked with intellectual disability. J Neural Transm (Vienna).

[65] Rosenfeld JA, Ballif BC, Torchia BS, Sahoo T, Ravnan JB, Schultz R (2010). Copy number variations associated with autism spectrum disorders contribute to a spectrum of neurodevelopmental disorders. Genet Med.

[66] Yamakawa H, Oyama S, Mitsuhashi H, Sasagawa N, Uchino S, Kohsaka S, Ishiura S (2007). Neuroligins 3 and 4X interact with syntrophin-gamma2, and the interactions are affected by autism-related mutations. Biochem Biophys Res Commun.

[67] Gilling M, Rasmussen HB, Calloe K, Sequeira AF, Baretto M, Oliveira G (2013). Dysfunction of the heteromeric KV7.3/KV7.5 potassium channel is associated with autism spectrum disorders. Front Genet.

[68] Peñagarikano O, Geschwind DH (2012). What does CNTNAP2 reveal about autism spectrum disorder?. Trends Mol Med.

[69] Chiocchetti AG, Kopp M, Waltes R, Haslinger D, Duketis E, Jarczok TA (2015). Variants of the CNTNAP2 5′ promoter as risk factors for autism spectrum disorders: a genetic and functional approach. Mol Psychiatry.

[70] Yamada T, Sakisaka T, Hisata S, Baba T, Takai Y (2005). RA-RhoGAP, Rap-activated Rho GTPase-activating protein implicated in neurite outgrowth through Rho. J Biol Chem..

[71] Amaral DG, Schumann CM, Nordahl CW (2008). Neuroanatomy of autism. Trends Neurosci.

[72] Patriquin MA, DeRamus T, Libero LE, Laird A, Kana RK (2016). Neuroanatomical and neurofunctional markers of social cognition in autism spectrum disorder. Hum Brain Mapp.

[73] Dickinson A, Jones M, Milne E (2016). Measuring neural excitation and inhibition in autism: different approaches, different findings and different interpretations. Brain Res.

[74] Bozzi Yuri, Provenzano Giovanni, Casarosa Simona (2017). Neurobiological bases of autism-epilepsy comorbidity: a focus on excitation/inhibition imbalance. European Journal of Neuroscience.

[75] Guglielmi L, Servettini I, Caramia M, Catacuzzeno L, Franciolini F, D'Adamo MC, Pessia M (2015). Update on the implication of potassium channels in autism: K(+) channelautism spectrum disorder. Front Cell Neurosci.

[76] Iourov IY, Vorsanova SG, Voinova VY, Yurov YB (2015). 3p22.1p21.31 microdeletion identifies CCK as Asperger syndrome candidate gene and shows the way for therapeutic strategies in chromosome imbalances. Mol Cytogenet.

[77] Varghese Merina, Keshav Neha, Jacot-Descombes Sarah, Warda Tahia, Wicinski Bridget, Dickstein Dara L., Harony-Nicolas Hala, De Rubeis Silvia, Drapeau Elodie, Buxbaum Joseph D., Hof Patrick R. (2017). Autism spectrum disorder: neuropathology and animal models. Acta Neuropathologica.

[78] Berkowicz SR, Featherby TJ, Qu Z, Giousoh A, Borg NA, Heng JI (2016). Brinp1(−/−) mice exhibit autism-like behaviour, altered memory, hyperactivity and increased parvalbumin-positive cortical interneuron density. Mol Autism.

[79] Quesnel-Vallières M, Dargaei Z, Irimia M, Gonatopoulos-Pournatzis T, Ip JY, Wu M (2016). Misregulation of an activity-dependent splicing network as a common mechanism underlying autism spectrum disorders. Mol Cell.

