# Supplementary material for: Loss of the Chr16p11.2 ASD candidate gene QPRT leads to aberrant neuronal differentiation in the SH-SY5Y neuronal cell model
Source: Mol Autism. 2018 Nov 6;9:56. doi: 10.1186/s13229-018-0239-z (PMC6220561; doi:10.1186/s13229-018-0239-z)
Supplement: Supplementary file 1 — Supplementary methods, supplementary results, supplementary table S1, supplementary figures S1-S6. (DOCX 555 kb) [file 13229_2018_239_MOESM1_ESM.docx]

Additional file 1

Loss of the Chr16p11.2 ASD candidate gene *QPRT* leads to aberrant neuronal differentiation in the SH-SY5Y neuronal cell model

Denise Haslinger^1^, Regina Waltes^1^, Afsheen Yousaf^1^, Silvia Lindlar^1^, Ines Schneider^2^, Chai K. Lim^3^, Meng-Miao Tsai^4^, Boyan K. Garvalov^4,5^, Amparo Acker-Palmer^6^, Nicolas Krezdorn^7^, Björn Rotter^7^, Till Acker^4^, Gilles J. Guillemin^3^, Simone Fulda^2^, Christine M. Freitag^1^, Andreas G. Chiocchetti^1^

^1^ Department of Child and Adolescent Psychiatry, Psychosomatics and Psychotherapy, University Hospital Frankfurt, JW Goethe University Frankfurt, Frankfurt am Main, Germany

^2^ Institute of Experimental Cancer Research in Pediatrics, Frankfurt am Main, Germany

^3^ Macquarie University, Faculty of Medicine and Health Sciences, Sydney, New South Wales, Australia

^4^ Neuropathology, University of Giessen, Giessen, Germany

^5^ Department of Microvascular Biology and Pathobiology, European Center for Angioscience (ECAS), Medical Faculty Mannheim, University of Heidelberg, Germany

^6^ Institute of Cell Biology and Neuroscience and Buchmann Institute for Molecular Life Sciences (BMLS), JW Goethe University of Frankfurt, Frankfurt am Main, Germany

^7^ GenXPro GmbH Frankfurt am Main

Index

[Supplementary methods 3](#_Toc525227404)

[Real-time RT-PCR: 3](#_Toc525227405)

[Protein extraction, SDS-PAGE and Western Blot: 3](#_Toc525227406)

[siRNA-mediated knock down (KD) of *QPRT*: 4](#_Toc525227407)

[CRISPR/Cas9-mediated knock out (KO) of *QPRT*: 5](#_Toc525227408)

[Metabolite analysis: 5](#_Toc525227409)

[Weighted gene co-expression network analysis (WGCNA): 7](#_Toc525227410)

[GO term enrichment analysis: 7](#_Toc525227411)

[Supplementary results 7](#_Toc525227412)

[Potential CRISPR/Cas9 off-targets for sgRNAs targeting *QPRT*: 7](#_Toc525227413)

[Guide sequence targeting QPRT Ex2.1 (introducing del268T in QPRT NM_014298): 8](#_Toc525227414)

[Guide sequence targeting QPRT Ex2.2 (introducing ins395A in QPRT NM_014298): 8](#_Toc525227415)

[Analysis of potential CRISPR/Cas9 off-targets for sgRNAs targeting *QPRT*: 9](#_Toc525227416)

[Supplementary tables 10](#_Toc525227417)

[Table S1: Primer and probes (universal probe library/UPL; Roche) used in this study 10](#_Toc525227418)

[Table S2: List of genes targeted by transcriptome analysis and genes differentially expressed in *QPRT-KO* cells (supplied as separate Excel file) 10](#_Toc525227419)

[Table S3: GO-terms identified for differentially regulated genes and genes of the *QPRT*-associated darkgrey module (supplied as separate Excel file) 11](#_Toc525227420)

[Supplementary figures 12](#_Toc525227421)

[Figure S1: 12](#_Toc525227422)

[Figure S2: 14](#_Toc525227423)

[Figure S3: 15](#_Toc525227424)

[Figure S4: 16](#_Toc525227425)

[Figure S5: 17](#_Toc525227426)

[Figure S6: 19](#_Toc525227427)

[Supplementary references 19](#_Toc525227428)

# Supplementary methods

## Real-time RT-PCR:

RNA was extracted using the GeneJet RNA purification kit (Fermentas) and transcribed into cDNA using the RevertAid H Minus cDNA Synthesis Kit (Thermo Fisher Scientific) according to the manufacturer’s protocol. Quantitative real-time PCR was performed using the StepOne Plus Real-Time PCR system (Applied Biosystems) making use of the Universal Probe Library system (UPL; Roche). One PCR reaction contained 10ng cDNA template, 1x ABsolute qPCR ROX Mix (Thermo Scientific), 200nM primer fwd, 200nM primer rev, 100nM UPL probe and PCR-grade H_2_O. For details on primer and probe combinations see Table S1.

## Protein extraction, SDS-PAGE and Western Blot:

For validation at protein level, protein was extracted using RIPA cell lysis buffer (50mM Tris pH 8, 150mM NaCl, 1% NP40, 0.5% sodium deoxycholate, 2mM MgCl_2_ freshly supplied with 0.1% SDS, 1µl/ml universal nuclease (Pierce) and proteinase inhibitor cocktail (PIC) cOmplete ULTRA Tablets Mini Easypack (Roche). Pellets were incubated in RIPA for 30 min on ice. The cell debris was pelleted via 14,000 x g centrifugation for 15 min at 4°C and supernatants containing the protein were collected and subjected to Pierce BCA Protein Assay Kit (Life Technologies) for measurement of concentration following the manufacturer’s protocol. 50-100µg of total protein lysate were mixed with protein loading dye (60mM Tris HCl pH6.8, 1% SDS, 5% glycerol, 0.01mg/ml bromophenol blue) and samples were adjusted to a final volume of 20µl with water. Protein samples were denatured at 95°C for 5 min. Gel electrophoresis was performed at 100 V until all samples entered the stacking gel (5% acrylamide/bis-acrylamide (37.5 : 1; 40%), 0.1% SDS, 125mM Tris HCl pH6.8, 0.1% APS, 0.1% TEMED) and migrated into the resolving gel (12% acrylamide, 0.1% SDS, 250mM Tris HCl pH8.8, 0.1% APS, 0.04% TEMED). Thereafter, voltage was increased up to 140V. Using a semidry approach, proteins were transferred to a nitrocellulose membrane with 1mA/cm^2^ for 1-2 hours. After blotting (blotting buffer 125mM Tris base, 1.25mM glycine, 0.1% SDS, 20% methanol), membranes were incubated in SuperBlock (Pierce) for 1 hour at room temperature. After application of first (mouse anti-QPRT antibody (Abcam), 1:1,000 in SuperBlock) and secondary antibody (anti-mouse IgG HRP conjugate; 1:5,000 in SuperBlock; Santa Cruz) membranes were washed three times with PBST (PBS supplemented with 0.1% Tween-20) for 10 min each. Proteins were detected using the ECL Prime Western Blot Detection Reagent and the Amersham Hyperfilm ECL (both GE Healthcare). Prior to application of controls, blots were stripped with 100mM β-mercaptoethanol, 2% SDS, 62.5mM Tris HCl pH6.7 for 30 min at 50°C and again blocked with SuperBlock for 1 h. β-Actin (Sigma; mouse, 1:10,000 in Superblock) or GAPDH (Santa Cruz; mouse; 1:2,500 in Superblock) was applied for 1 hour at room temperature. The subsequent steps including secondary antibody and visualization were performed as described above.

## siRNA-mediated knock down (KD) of *QPRT*:

For siRNA transfection we used three different Silencer® Select Pre-designed siRNAs for QPRT, i.e.

“siQ1”: s23898 (Lot# ASO20MTK), “siQ2”: s23899 (Lot# ASO20MTJ) and “siQ3”: s23900 (Lot# ASO20MTI); and the Silencer® Select Negative Control 1 (all Ambion, Thermo Fisher).

Lyophilized pellets were diluted in nuclease-free water (Ambion, Thermo Fisher) and stored at -20°C as 20µM stocks. The final concentration for transfection was 5nM.

## CRISPR/Cas9-mediated knock out (KO) of *QPRT*:

sgRNAs were designed using an online tool [1]:

Ex.2.1 5’ CACCGCAGTTGAGTTGGGTAAATA 3’

3’ CGTCAACTCAACCCATTTATCAAA 5’

Ex.2.2 5’ CACCGCAGCGGGCCAGCGTGTTGA 3’

3’ CGTCGCCCGGTCGCACAACTCAAA 5’

Oligos were ordered from Sigma Aldrich and cloned into pSpCas9(BB)-2A-Puro (PX459) V2.0 (Plasmid #62988; Addgene).

Primer used for Sanger-Sequencing:

Ex.2.1/Ex.2.2_fw GAGAGGCAGCCAAACTCAA

Ex.2.1/Ex.2.2_rv ACCAGGAGCCCATACTTCT

## Metabolite analysis:

Ultra-high-performance liquid chromatography (UHPLC) and gas chromatography-mass spectrometry (GC/MS) were performed as described previously [2, 3]. Prior to analysis, culture media and serum samples were deproteinized with trichloroacetic acid at a final concentration of 5% (w+v) in equal volume. Samples were incubated for 5 min, vortexed and then centrifuged (4°C) for 10 min at 12,000 rpm. Supernatant was extracted, filtered with syringe filters (0.22μm) and subsequently injected into analyzers.

Concurrent quantification of tryptophan (TRP), kynurenine (KYN), 3-hydroxykynurenine (3-HK), 3-hydroxyanthranilic acid (3HAA), and anthranilic acid (AA) was carried out in accordance to [2]. An Agilent 1290 infinity ultra-high-performance liquid chromatography (UHPLC) system coupled with temperature controlled autosampler and column compartment, diode array detector and fluorescence detector were used for the analysis of these metabolites with a 20μL sample injection volume. Separation of metabolites was performed under stable temperature of 38°C for 12 min, using 0.1mM sodium acetate (pH4.65) as mobile phase, with an isocratic flow rate of 0.75ml/min in an Agilent Eclipse Plus C18 reverse-phase column (2.1mm x 150mm, 1.8μm particle size). 3HK and KYN were detected using an UV wavelength of 365nM with a retention time of 1.2 and 3.1 min, respectively. TRP, 3HAA and AA were detected using fluorescence intensity set at Ex/Em wavelength of 280/438 nm for TRP and 320/438 nm for 3HAA and AA. Mixed standards of all metabolites were used for a six-point calibration curve in order to interpolate the quantity of the sample readout. Agilent OpenLAB CDS Chemstation (Edition C.01.04) was used to analyze the chromatograms. The inter- and intra-assay coefficient of variation was within the acceptable range of 3-7%.

Kynurenic acid (KA) detection was carried out using UHPLC with a gradient mobile phase comprising 50mM sodium acetate buffer spiked with 25mM zinc acetate (dihydrate) to enhance fluorescence intensity and 2.25% acetonitrile as organic modifier (solvent A), and 10% acetonitrile (solvent B). Each sample (10μL) was injected into a Poroshell RRHT C-18, 1.8μm 2.1 × 100mm column (Agilent Technologies, Inc, Santa Clara, CA) maintained at 38°C for a 12 min run time at a unison flowrate of 0.75ml/min. The gradient elution comprised 100% solvent A for 3 min and then 50% solvent A and 50% solvent B for 2 min, followed by 100% solvent B for 2 min and 100% solvent A (run time of 10 min). This gradient ensured sufficient time for KA retention while minimizing potential build-up of pressure due to precipitation of the high salt buffer. Fluorescence was used for detection of KA (excitation and emission wavelengths of 344nm and 388nm, respectively with a retention time of 1.5 min). Agilent OpenLAB CDS ChemStation (Edition C.01.04) was used to analyze the chromatograms.

Picolinic acid (PIC) and quinolinic acid (QUIN) were detected using gas chromatography–mass spectrometry (GC/MS) as previously described in [3]. Separation of PIC and QUIN were achieved using a DB-5MS column, 0.25μm film thickness, 0.25mm × 30m capillary column (Agilent Technologies, Inc, Santa Clara, CA) with a 1µL injection volume of the derivatized sample. Quantification of PIC and QUIN concentrations were analysed using Agilent GC/MSD ChemStation software (Edition 02.02.1431) and interpolated from the established six-point calibration curves based on the abundance count ratio of the metabolites to their corresponding deuterated internal standards within each standard and sample. The intra- and inter-assay coefficient of variability was within the acceptable range (4–8% for UHPLC assays, 7–10% for GC/MS assays) calculated from the repeated measures of the metabolite standards incorporated during the sequence run.

## Weighted gene co-expression network analysis (WGCNA):

WGCNA was performed on the normalized (DESeq2) and log2 transformed read counts of all expression data with non-zero variance. The signed adjacency is defined as a=(0.5*(1+cor))^stp, where stp is the soft thresholding power chosen to obtain a scale free network. Here, a stp of 18 resulted in a scale free network based on a regression coefficient R² > 0.8 from a scale free topology modeling approach. Modules (i.e. genes with high topological overlap) were defined using hierarchical clustering (distance=distance of topological overlap, method=average linkage) and the ‘cutreeDynamic’ algorithm as published, with a minimum module size of N = 30 [4]. Modules were clustered (distance=Euclidean, linkage=complete) based on correlation distance between their eigenvalues (eigengene values) and merged below a cluster height=0.1 (module dissimilarity).

## GO term enrichment analysis:

GO term enrichment was performed with the ‘topGO’ package using the *weight01* algorithm, where the significance of a GO term is weighted by the enrichment score of related GO terms in combination with a bottom up elimination algorithm [5] increasing efficiency in detecting relevant associated terms. Individual tests are not independent and therefore multiple testing does not apply. P-values extracted thus can be considered as corrected for multiple testing. The gene universe was defined as the 32,739 transcripts expressed.

# Supplementary results

## Potential CRISPR/Cas9 off-targets for sgRNAs targeting *QPRT*:

For both sgRNAs the highest (i.e. worst) off target score was 0.9. Here, in both cases this meant that 4 mismatches (MM) were occurring between the sgRNA and the potential off-target sequence.

The score is calculated from (i) the number of MM between sgRNA and off-target sequence, (ii) the position of the MM, as those close to the PAM sequence are more deleterious and (iii) the mean pairwise distance between MM, as two or more MM close to each other have a higher disruptive effect on guide-DNA interaction (http://crispr.mit.edu).

### Guide sequence targeting QPRT Ex2.1 (introducing del268T in QPRT NM_014298):

GCAGTTGAGTTGGGTAAATATGG

on-target locus: chr16:-29706155; quality score: 64

number of off-target sites: 224 (16 are in genes)

| **sequence** | **score** | **mismatches** | **UCSC gene** | **Gene name** | **locus** | **expr eCtrl vs KO** |
| --- | --- | --- | --- | --- | --- | --- |
| ACAGTTTCGGTGGGTAAATAAGG | 0.9 | 4MMs [1:7:8:10] | NM_001257268 | RD3L | chr14:-104408546 | Not sig |
| GCAGCTGAATGGGGTAAATATGG | 0.9 | 3MMs [5:9:11] | NM_001359 | DECR1 | chr8:+91054961 | Not sig |
| GGAGTGAAGATGGGTAAATACGG | 0.5 | 4MMs [2:6:7:10] | NM_004644 | AP3B2 | chr15:+83345404 | Not sig |
| GCAGTAGACATTGGTAAATATGG | 0.2 | 4MMs [6:9:10:12] | NM_012203 | GRHPR | chr9:+37426473 | Not sig |
| ACTGTTGAGTTAGGAAAATATGG | 0.2 | 4MMs [1:3:12:15] | NM_001139510 | ECHDC1 | chr6:-127609970 | Not sig |
| GCATTTCAGTGGGGAAAATACAG | 0.2 | 4MMs [4:7:11:15] | NR_002808 | ITPK1-AS1 | chr14:-93534420 | Not sig |
| CCAGATGAGTTGTGTGAATAAGG | 0.1 | 4MMs [1:5:13:16] | NM_001257230 | ALG13 | chrX:+110970861 | Not sig |
| GCAGTGGGTTTGGGTCAATATGG | 0.1 | 4MMs [6:8:9:16] | NM_001802 | CDR2 | chr16:-22360921 | Not sig |
| TGAGTTGAGTTGGGTGAAGAGGG | 0.1 | 4MMs [1:2:16:19] | NM_001143983 | CHRDL1 | chrX:+110002819 | Not sig |
| GCAGATGAGGTGGGTAAGAAGGG | 0.1 | 4MMs [5:10:18:19] | NM_178817 | MRAP | chr21:+33679040 | Not sig |
| GCAGGTGAGGTGGTTATATAAGG | 0.1 | 4MMs [5:10:14:17] | NM_017697 | ESRP1 | chr8:+95686829 | Not sig |
| GCAGTTGGGTTGGGTATACCGAG | 0.1 | 4MMs [8:17:19:20] | NM_005407 | SALL2 | chr14:+21993822 | Not sig |
| GCAGTTGATTATGATAAATACAG | 0.0 | 4MMs [9:11:12:14] | NM_001369 | DNAH5 | chr5:-13737698 | Not sig |
| GCAGTTGAGTTAGGGAGACAGAG | 0.0 | 4MMs [12:15:17:19] | NM_022648 | TNS1 | chr2:+218668778 | Not sig |
| GCAGTTGAGGTGGGCAAGAAAAG | 0.0 | 4MMs [10:15:18:19] | NM_023073 | C5orf42 | chr5:-37198810 | Not sig |

### Guide sequence targeting QPRT Ex2.2 (introducing ins395A in QPRT NM_014298):

GCAGCGGGCCAGCGTGTTGAGGG

on-target locus: chr16:-29706281; quality score: 81

number of off-target sites: 134 (27 are in genes)

| **sequence** | **score** | **mismatches** | **UCSC gene** | **Gene name** | **locus** | **expr eCtrl vs KO** |
| --- | --- | --- | --- | --- | --- | --- |
| GCTCAGTGCCAGCGTGTTGATGG | 0.9 | 4MMs [3:4:5:7] | NM_015456 | NELFB | chr9:-140160356 | Not sig |
| CCAGAGGGCTAGCGCGTTGAGGG | 0.4 | 4MMs [1:5:10:15] | NM_001142961 | LPAR5 | chr12:+6730278 | Not sig |
| GAGGCGGGCCTGAGTGTTGACGG | 0.3 | 4MMs [2:3:11:13] | NM_024341 | ZNF557 | chr19:-7069391 | Not sig |
| GAAGAGGGCCAGAGTGCTGAGAG | 0.2 | 4MMs [2:5:13:17] | NM_024712 | ELMO3 | chr16:-67236979 | Not sig |
| GCTGAGGACCAGCTTGTTGAAGG | 0.2 | 4MMs [3:5:8:14] | NM_015529 | MOXD1 | chr6:+132618972 | Not sig |
| GCAGGGGGCGCGCGGGTTGAGGG | 0.2 | 4MMs [5:10:11:15] | NM_001243531 | UBE2Q2L | chr15:+84841340 | Not sig |
| TCAGCCGGCCATCGTGGTGAAGG | 0.2 | 4MMs [1:6:12:17] | NM_012168 | FBXO2 | chr1:-11709835 | Not sig |
| GCAGCTGGCCAGGGGGTTGATGG | 0.2 | 3MMs [6:13:15] | NM_014010 | ASTN2 | chr9:+119737526 | Not sig |
| CCTGCGGGCCAGCGAGCTGACGG | 0.2 | 4MMs [1:3:15:17] | NM_013291 | CPSF1 | chr8:-145619729 | Not sig |
| GCAGCGGGCGAGGGTCTTGATAG | 0.2 | 3MMs [10:13:16] | NM_022124 | CDH23 | chr10:+73494196 | Not sig |
| GCAGCTGACCAGAGTGATGACGG | 0.1 | 4MMs [6:8:13:17] | NM_006045 | ATP9A | chr20:+50217820 | Not sig |
| GCTGCAGCCCAGCATGTTGAAGG | 0.1 | 4MMs [3:6:8:14] | NM_153330 | DNAJB8 | chr3:+128181616 | Not sig |
| TCATCGGGCCAGCGTCTTGCCAG | 0.1 | 4MMs [1:4:16:20] | NR_024563 | LOC100130238 | chr12:-132852055 | Not sig |
| TCAGGGGGCCAGGCTGTTGAAGG | 0.1 | 4MMs [1:5:13:14] | NM_031282 | FCRL4 | chr1:-157556180 | Not sig |
| CCAGCTGGCCAGTGTGTGGATGG | 0.1 | 4MMs [1:6:13:18] | NM_001252043 | TAOK2 | chr16:-29999115 | Not sig |
| GCAGCGAGCAAGCGTGTGCAGGG | 0.1 | 4MMs [7:10:18:19] | NM_018289 | VPS53 | chr17:+456523 | Not sig |
| GCTGCTGGCCAGCTTGTTAAAGG | 0.0 | 4MMs [3:6:14:19] | NM_014856 | DENND4B | chr1:+153902867 | increased |
| GCTGCGGGCCAGCGTCTTATGGG | 0.0 | 4MMs [3:16:19:20] | NM_001200049 | CFAP46 | chr10:-134624087 | Not sig |
| GCAGCTGGACAGCGTCATGAGAG | 0.0 | 4MMs [6:9:16:17] | NM_053052 | SNAP47 | chr1:+227935870 | increased |
| GCAGCGGCCCAGGATGTTGGCGG | 0.0 | 4MMs [8:13:14:20] | NM_018364 | RSBN1 | chr1:+114354657 | Not sig |
| GCAGGGGGGCAGCTGGTTGAGAG | 0.0 | 4MMs [5:9:14:15] | NM_002646 | PIK3C2B | chr1:+204426884 | Not sig |
| GCACCGGGCCAGCGAGGGGATGG | 0.0 | 4MMs [4:15:17:18] | NM_001130413 | SCNN1D | chr1:+1216863 | increased |
| GCAGCAGGCCGGCATGGTGACAG | 0.0 | 4MMs [6:11:14:17] | NM_002562 | P2RX7 | chr12:-121570761 | Not sig |
| GCAGCTGGCCAACGTGTGTAGAG | 0.0 | 4MMs [6:12:18:19] | NR_003676 | OTOAP1 | chr16:+22588035 | Not sig |
| GCAGCGGGACTGCCTGGTGAGGG | 0.0 | 4MMs [9:11:14:17] | NM_001195733 | PIP5K1C | chr19:-3653276 | Not sig |
| GCACCGGGCCAGCAAGCTGACGG | 0.0 | 4MMs [4:14:15:17] | NM_014780 | CUL7 | chr6:-43014057 | Not sig |

## Analysis of potential CRISPR/Cas9 off-targets for sgRNAs targeting *QPRT*:

We checked the 43 potential genic off-targets (16+27) in the RNA-Seq (MACE) data of the KO, empty control and WT cells. Of those, 30 genes harboring potential off-target sequences were expressed in the MACE analysis. After omitting genes differentially expressed between eCtrl and WT, only three of the potential off-targets were differentially expressed in the respective KO (SNAP47: FDR = 0.007, log2FC = 0.29; DENND4B: FDR = 0.001, log2FC = 0.38; SCNN1D: FDR = 3.21-E04, log2FC= 0.95). Since all significant effects show an increase of expression in the KO, an unspecific knock out is unlikely. In addition, we only analyzed effects that are replicated in both KO cell lines thus we consider these effects as negligible.

# Supplementary tables

## Table S1: Primer and probes (universal probe library/UPL; Roche) used in this study

| **Gene** | **RefSeq-Nr.** | **Forward** | **Reverse** | **UPL probe** |
| --- | --- | --- | --- | --- |
| *ARHGAP20* | NM_020809.3 | tgagatgtgacactagagagaatgc | ctcatttagctcattttccaagc | 63 |
| *BRINP1* | NM_014618.2 | aaaagtacggcacccacct | catatacatggtcaaagcctcct | 5 |
| *CCK* | NM_000729.5 | ctttcactgacccagaacactg | cggttgaagtggctcctg | 80 |
| *COX17* | NM_005694.1 | aagatgccgggtctggtt | ttcttctcctttctcgatgataca | 2 |
| *COX17P1* | ENST00000535895 | caagtgcagcagggcttc | aaagatgccgggtctggt | 2 |
| *CUX2* | NM_015267.3 | cgtcatcaactggttccaca | ggatcaaggtctggctcatc | 22 |
| *GAPDH* | [NM_002046.3](https://qpcr.probefinder.com/showsequence.jsp?seqNo=149141953) | agccacatcgctcagacac | gcccaatacgaccaaatcc | 60 |
| *GUCA1A* | NM_000409.4 | caggacctcgagcagtctct | gagtctccactaaatccttgcag | 66 |
| *GUSB* | [NM_000181.3](https://qpcr.probefinder.com/showsequence.jsp?seqNo=669851417) | cgccctgcctatctgtattc | gatgaggaactggctcttgg | 5 |
| *KCNQ3* | NM_004519.3 | aagggtcagcattcaccttc | tgatgtggatggtctggcta | 14 |
| *LINC01760* | NR_135588 .1 | aggcaacttactagactaagggtga | tgcatttctcaaagcagacaa | 4 |
| *NLGN3* | NM_018977.3 | cccaacgaagactgtctctacc | cctcgccctgtttcttagc | 88 |
| *NMNAT2* | NM_015039.3 | acacctggcagacgacct | ggtgtgttgacattggagagg | 71 |
| *PSMD7* | NM_002811.4 | cacgtgaccagtgaaattgg | ccaccgtcgtgtctttgata | 6 |
| *QPRT* | NM_014298.3 | tggaagtggaatgcagcag | gaactgggccttcagcac | 82 |
| *SNTG2* | NM_018968.3 | ttttcaacgtggagcttgg | gctgcacatgtatgttctgga | 7 |
| *SRRM4* | NM_194286.3 | gctcctcctatgccagcac | gagagcgggagtaggacctt | 65 |
| *VSTM2A* | NM_182546.3 | ctggagatccaatggtggtt | ctgtccgggtctctgtcg | 39 |

## Table S2: List of genes targeted by transcriptome analysis and genes differentially expressed in *QPRT-KO* cells (supplied as separate Excel file)

Descriptions: del268T: CRISPR/Cas9 induced mutation (deletion of one nucleotide) in exon 2 of QPRT; ins395A: CRISPR/Cas9 induced mutation (insertion of one nucleotide) in exon 2 of QPRT; eCtrl: Control cell line with empty CRISPR/Cas9 control vector; WT: Wild type SH-SY5Y cell line, untreated; FDR: False discovery rate; log2FC: log2 fold change; Module membership: Respective module of co-regulated gene set as identified via WGCNA.

## Table S3: GO-terms identified for differentially regulated genes and genes of the *QPRT*-associated darkgrey module (supplied as separate Excel file)

Description: GO.ID: Gene ontology identity; Term: Name of GO-term; Annotated: Number of genes annotated with the respective GO-term; Significant: Number of genes within respective gene set and annotated with respective GO-term; Expected: Number of genes in gene set expected to be GO-term annotated; weight01: Significance of GO-term weighted by the enrichment score of related GO-terms in combination with a bottom up elimination algorithm [5]; Genes: Genes within respective module; Set: Set of differentially expressed genes or genes of a module.

# Supplementary figures

Supplementary figure 1


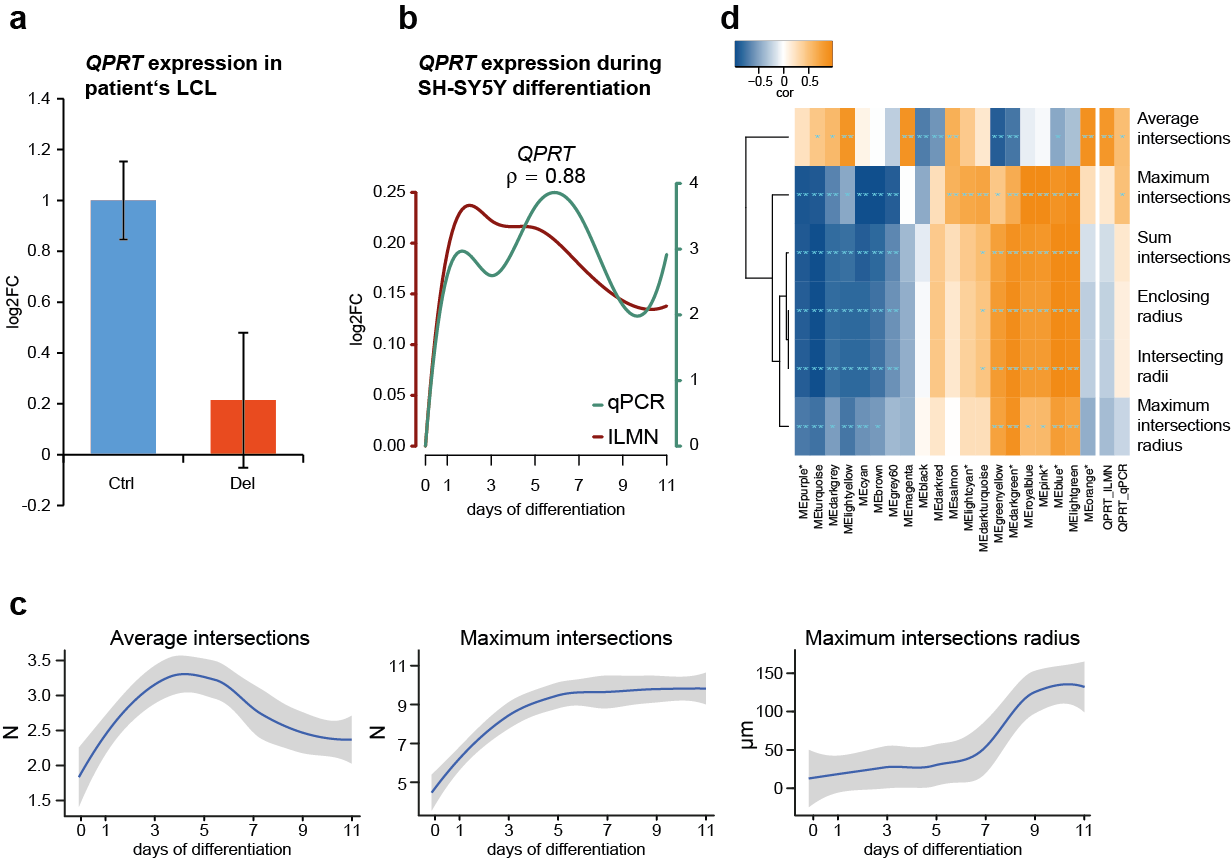


## Figure S1:

(a) ***QPRT* expression in lymphoblastoid cell lines of a 16p11.2 deletion carrier and control cell lines.** *QPRT* was expressed in a gene dosage dependent manner at mRNA level (16p11.2 deletion carrier compared to controls; logFC = -0.68, *p* = 0.014). (b) ***QPRT* expression during SH-SY5Y differentiation.** Expression of *QPRT* during neuronal differentiation correlated between microarray (ILMN) and real-time RT-PCR (qPCR; ρ = 0.88, *p* = 0.0098). (c) **Morphological Sholl analysis of wild type SH-SY5Y cells during neuronal differentiation.** Differentiated cells developed a stereotypical multipolar neuronal morphology, reaching the maximum number of neurites after 5 days of differentiation (maximum intersections) followed by neurite outgrowth (maximum intersections radius). (d) **Correlation heatmap of SH-SY5Y gene expression and morphology during neuronal differentiation.** The expression of *QPRT* as well as of its co-regulated gene-set correlated highly significantly with the morphological parameter average intersections (QPRT_ILMN: ρ = 0.86, FDR = 8.16E-05; QPRT_qPCR: ρ = 0.54, FDR = 0.020; MEorange: ρ = 0.93, FDR = 1.71E-07), describing the neuritic complexity of a cell. Asterisks mark the module eigengene (ME) of modules enriched for ASD risk genes as defined by AutismKB [7] (N=3,050 non-syndromic and syndromic risk genes).

Enclosing radius: Outer radius intersecting the cell; indicating the cell’s total size. Maximum intersections: Maximum number of intersections. Intersecting radii: Number of radii intersecting one cell. Sum intersections: Sum of all intersections per cell. Maximum intersections radius: Radial distance of the maximum number of intersections from the cell body; indicates at which distance from the cell soma the highest complexity can be found. Average intersections: Sum of all intersections divided by the number of intersecting radii; reflects the complexity of the neurites.

Supplementary figure 2


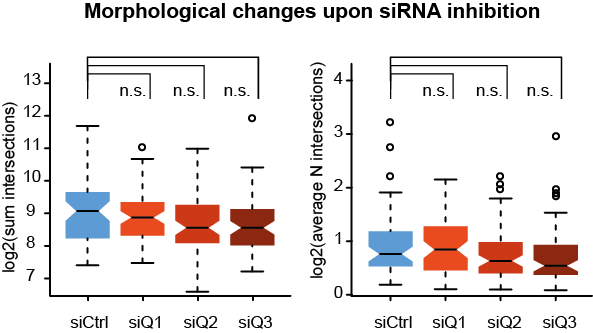


## Figure S2:

**siRNA mediated knockdown (KD) of *QPRT*.** While we observed significant reductions of the maximum intersections radius (Figure 1A), KD cells did not show significant changes of the overall sum of intersections (*p* siQ1: 0.396, siQ2: 0.064, siQ3: 0.064; Means[SD] siCtrl: 726.43[650.78]; siQ1: 553.86[356.01]; siQ2: 470.85[307.45]; siQ3: 516.05[505.19]) or the mean intersections per cell (*p* siQ1: 0.957, siQ2: 0.132, siQ3: 0.054; Means[SD] siCtrl: 2.14[1.37]; siQ1: 1.99[0.77]; siQ2: 1.82[0.78]; siQ3: 1.76[0.94]).

Supplementary figure 3


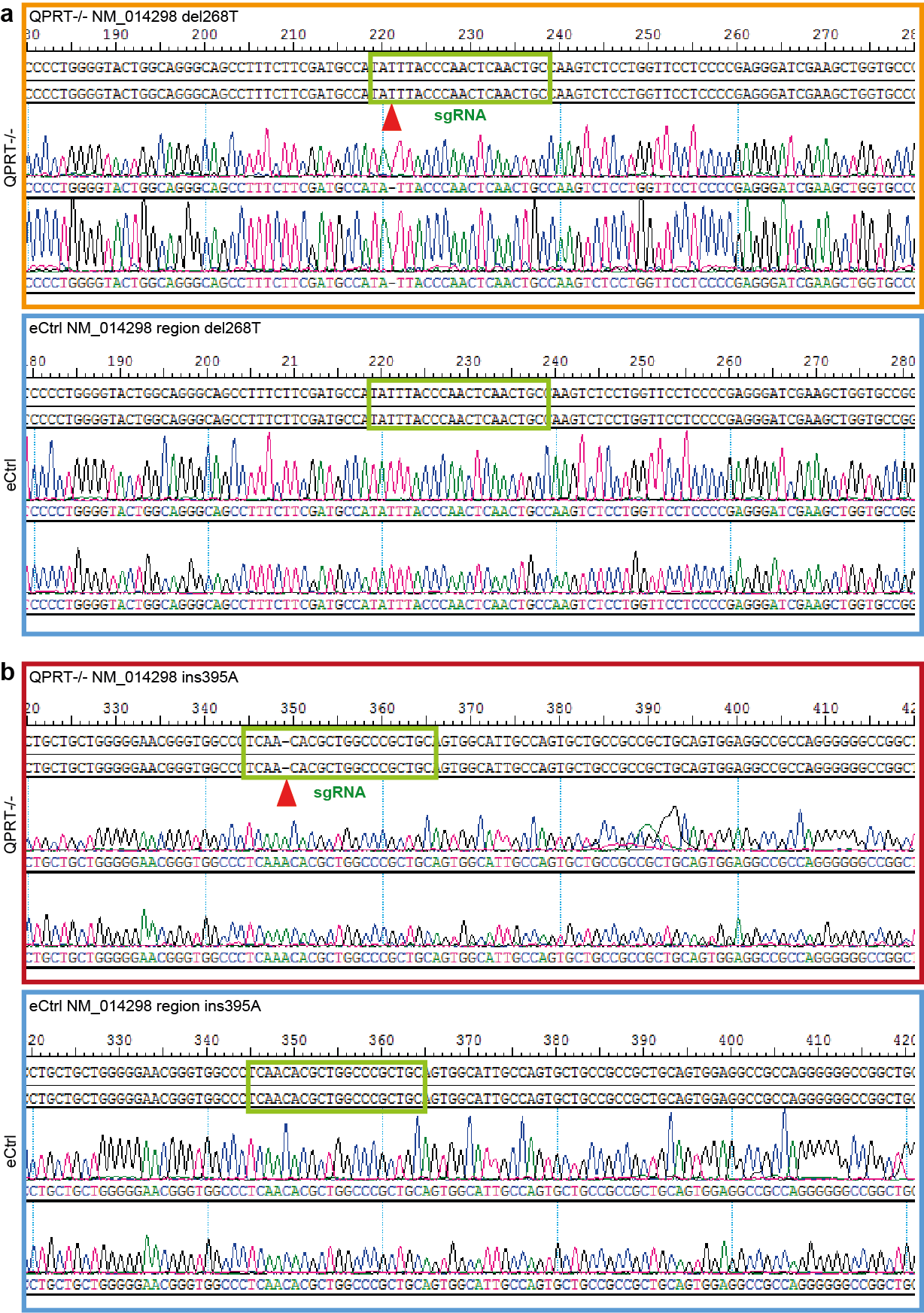


## Figure S3:

**Sequencing of *QPRT-KO* cell lines.** Results confirm the generation of indels (red triangles) leading to frameshifts. (a) del268T (*QPRT^-/-^* NM_014298 del268T; Ex2.1; premature stop codon at position 580) and (b) ins395A (*QPRT^-/-^* NM_014298 ins395A; Ex2.2; premature stop codon at position 593). Sequencing of the eCtrl (empty control vector) for both of the targeting regions does not show any mutations.

Supplementary figure 4


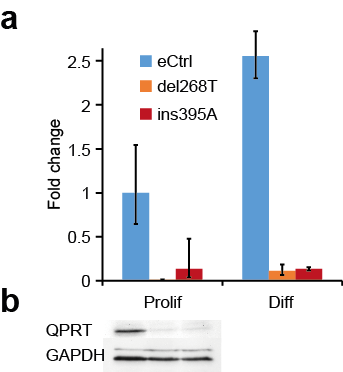


## Figure S4:

***QPRT* expression in generated KO and control cell lines.** Loss of *QPRT* was confirmed for both del268T and ins395A on RNA level (top) after 2 days of differentiation (all samples compared to proliferating empty control eCtrl; del268T: below detection limit; ins395A: FC = 0.17, *p* = 0.046; diff empty control: FC = 2.74, *p* = 0.009; diff del268T: FC = 0.13, *p* = 0.003; diff ins395A: FC = 0.15, *p* = 0.003). For proliferating cells, KO was furthermore descriptively confirmed at protein level (bottom).

Supplementary figure 5


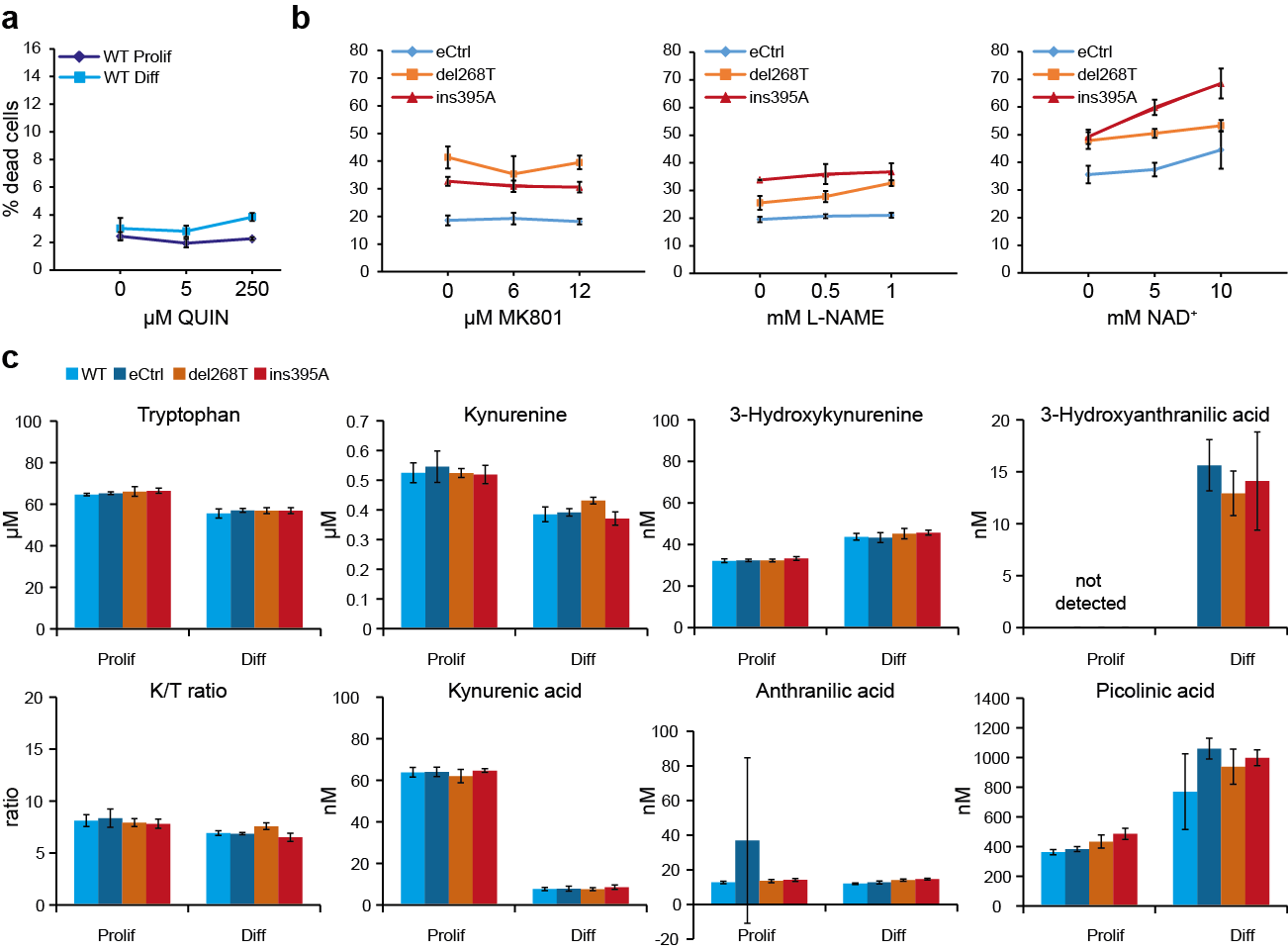


## Figure S5:

(a) **Application of quinolinic acid (QUIN) to proliferating and differentiating wild type (WT) SH-SY5Y cells.** Propidium iodide (PI) assays did not show a significant increase of cell death upon QUIN application during proliferation (all *p* > 0.3 for 5 µM and 250 µM QUIN) or 3 days of differentiation (all *p* > 0.3 for 5 µM and 250 µM QUIN). (b) **Rescue experiments of *QPRT-KO* induced cell death.** Neither the NMDA-R antagonist MK801 (both *QPRT-KO* compared to control: FC > 1.7, *p* < 3.9E-03 for 0 µM; FC > 1.6, *p* < 0.04 for 6 µM; FC > 1.6, *p* < 1.5E-03 for 12 µM) nor the NOS1 inhibitor L-NAME (both *QPRT-KO* compared to control: FC > 1.3, *p* < 0.05 for 0 mM; FC > 1.3, *p* < 0.02 for 0.5 mM; FC > 1.5, *p* < 0.01 for 1 mM) could rescue the increased cell death. Furthermore, application of the more downstream target NAD^+^ (both *QPRT-KO* compared to control: FC > 1.3, *p* < 9E-03 for 0 mM; FC > 1.3, *p* < 1.6E-03, for 5 mM; FC > 1.1, *p* < 0.1 for 10 mM) could not rescue *QPRT-KO*-induced cell death after 3 days of differentiation. (c) **Metabolite analysis of *QPRT-KO* cell lines and controls.** TRP, KYN, KA, 3HK, AA and PIC were measurable in all tested samples. QUIN could not be detected in any of the samples, while 3HAA could be detected in the 3 days differentiated (eCtrl and both of the KO) cell lines only. However, no significant changes could be observed in any of the metabolites when comparing both of the *QPRT-KO* cell lines to the control cell lines (all *p* > 0.1).
Abbreviations: TRP: tryptophan; KYN: kynurenine; KA: kynurenic acid; 3HK: 3-hydroxykunerine; AA: anthranilic acid; PIC: picolinic acid; QUIN: quinolinic acid; 3HAA: 3-hydroxyanthranilic acid.

Supplementary figure 6


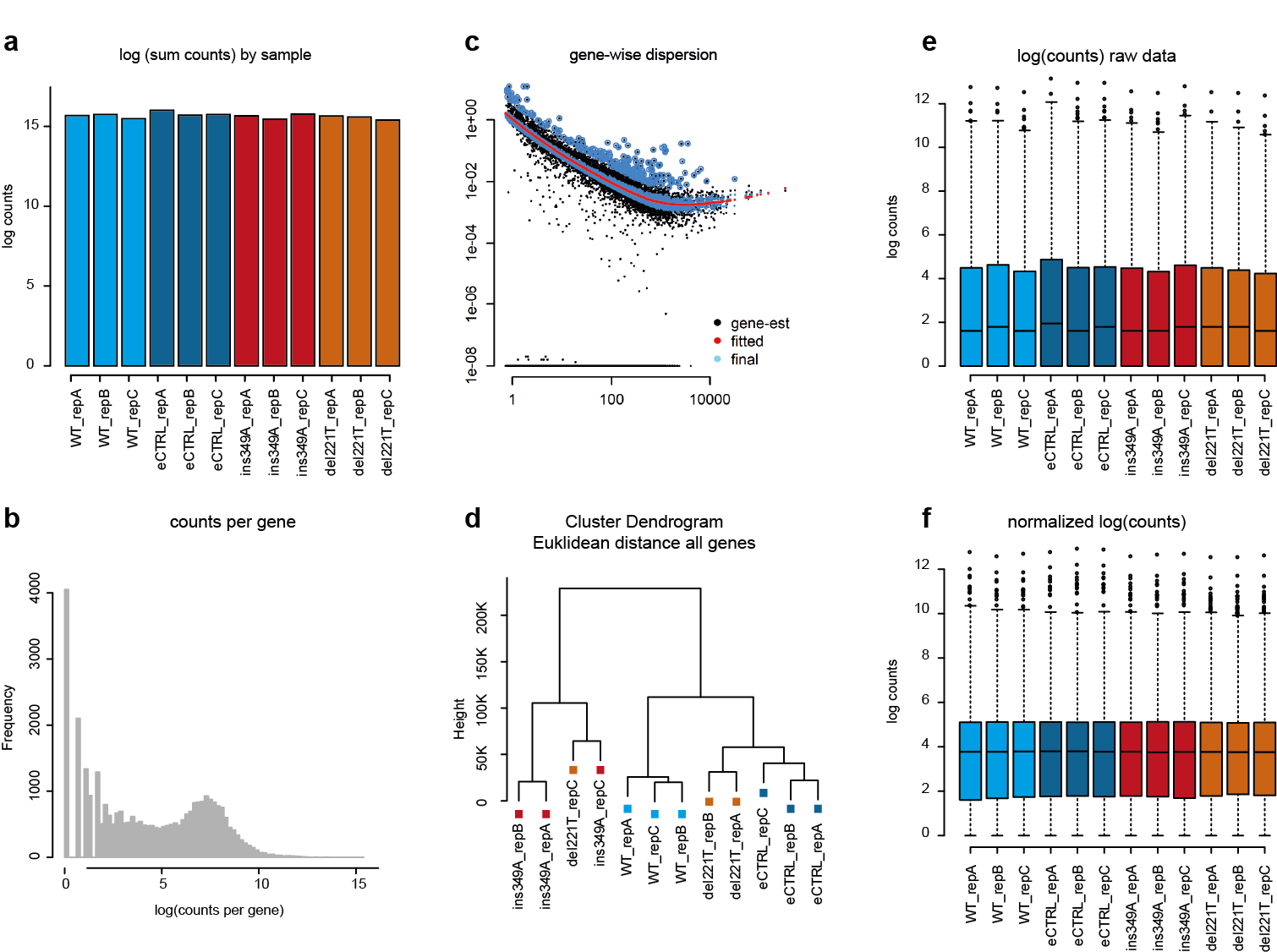


## Figure S6:

**Transcriptome quality check analyses.** (a) Total counts per sample are equally distributed across groups (ANOVA p-value = 0.223). (b) Total read counts per gene, no genes have been filtered for downstream analysis a-priori. However, DESeq2 analysis includes strict filtering of low-read genes and samples. (c) Dispersion estimates calculated in DESeq2 based on a numerical integration (option fitType="locfit" is used to stabilize variance based on the linear model including the group as independent variable. (d) Hierarchical cluster analysis has been performed to visually inspect the sample for technical outliers. All detected genes have been selected to calculate the Euclidean distance, the cluster-dendrogram was calculated using the ward.2 algorithm. The result [not shown] was comparable when using the top 2,000 genes based on variance only. Not-normalized (e) and normalized (f) count distribution per sample. Normalized counts were used for WGCN analysis only, while DESeq2 data requires raw count data.

Supplementary references

1. CRISPR design. <http://crispr.mit.edu/>. Accessed 30 Apr 2018.

2. Lim CK, Bilgin A, Lovejoy DB, Tan V, Bustamante S, Taylor BV, et al. Kynurenine pathway metabolomics predicts and provides mechanistic insight into multiple sclerosis progression. Sci Rep. 2017;7:41473. doi:10.1038/srep41473.

3. Lim CK, Essa MM, Paula Martins R de, Lovejoy DB, Bilgin AA, Waly MI, et al. Altered kynurenine pathway metabolism in autism: Implication for immune-induced glutamatergic activity. Autism Res 2015. doi:10.1002/aur.1565.

4. Langfelder P, Horvath S. Eigengene networks for studying the relationships between co-expression modules. BMC Syst Biol. 2007;1:54. doi:10.1186/1752-0509-1-54.

5. Alexa A, Rahnenführer J, Lengauer T. Improved scoring of functional groups from gene expression data by decorrelating GO graph structure. Bioinformatics. 2006;22:1600–7. doi:10.1093/bioinformatics/btl140.

6. Chiocchetti AG, Haslinger D, Stein JL, La Torre-Ubieta L de, Cocchi E, Rothamel T, et al. Transcriptomic signatures of neuronal differentiation and their association with risk genes for autism spectrum and related neuropsychiatric disorders. Transl Psychiatry. 2016;6:e864. doi:10.1038/tp.2016.119.

7. AutismKB. <http://autismkb.cbi.pku.edu.cn/>. Accessed 30 Apr 2018.
